# Supplementary material for: A three enzyme system to generate the Strychnos alkaloid scaffold from a central biosynthetic intermediate
Source: Nat Commun. 2017 Aug 22;8:316. doi: 10.1038/s41467-017-00154-x (PMC5566405; doi:10.1038/s41467-017-00154-x)
Supplement: Supplementary file 1 — Supplementary Information [file 41467_2017_154_MOESM1_ESM.pdf]

File Name: Supplementary Information
Description: Supplementary Figures

File Name: Supplementary Data 1
Description: SGD-targeted correlation analysis. Pearson Correlation Coefficients were calculated for SGD (SRR648705_TR21994_c0_g4_i4_len=2338) with all other transcripts in the CDF97 transcriptome assembly. Correlations were calculated using the following samples: ERR1512369, ERR1512370, ERR1512371, ERR1512372, ERR1512373, ERR1512374, ERR1512375, ERR1512376, ERR1512377, SRR1144633, SRR1144634, SRR1271857, SRR1271858, SRR1271859, SRR342017, SRR342019, SRR342022, SRR342023, SRR646572, SRR646596, SRR646604, SRR648705, SRR648707, SRR924147, SRR924148. Only transcripts having a PCC>0.6 are shown. Homologies were searched against Uniprot100 database with BlastX and Pfam domains against PfamA with Hmmerscan. Pathway and keyword information were retrieved from Uniprot annotation system.

File Name: Peer Review File
Description:

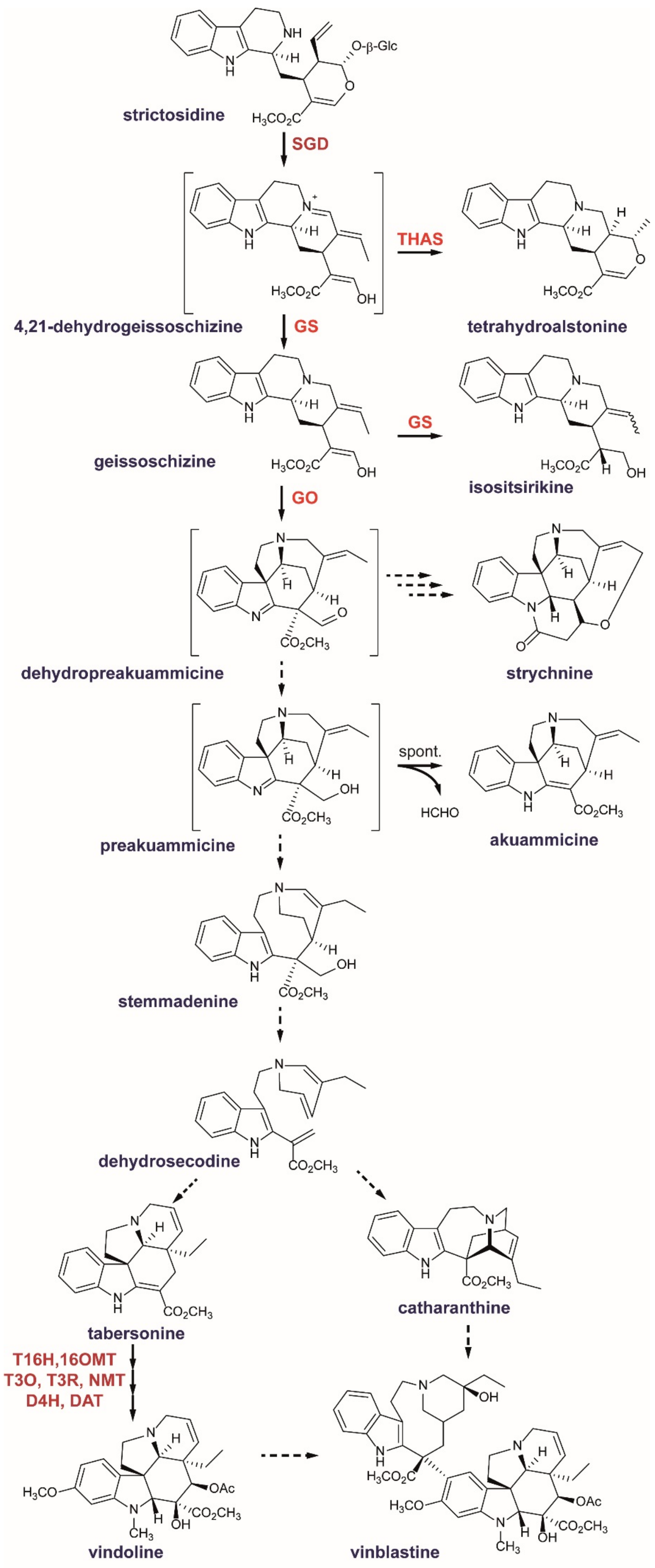
strictosidine
O-β-Glc
NH
H
O
H3CO2C
SGD
4,21-dehydrogeissoschizine
THAS
tetrahydroalstonine
GS
geissoschizine
GS
isositsirikine
GO
dehydropreakuammicine
strychnine
preakuammicine
spont.
HCHO
akuammicine
stemmadenine
dehydrosecodine
tabersonine
catharanthine
T16H,16OMT
T3O, T3R, NMT
D4H, DAT
vindoline
vinblastine

Supplementary Figure 1. Schematic overview of monoterpene indole alkaloids (MIAs) metabolism focusing on biosynthesis of anticancer drug vinblastine and notorious poison strychnine, depicting the central role of geissoschizine oxidation and rearrangement to the *strychnos* structural scaffold. According to literature data, dehydropreakuammicine is believed to be the precursor to strychnine biosynthesis, while preakuammicine is hypothesized to serve as common precursor to tabersonine and catharanthine, the precursors to vinblastine. Dashed arrows denote reactions by unknown enzymes, while multiple arrows denote multiple reactions. Abbreviations: SGD, strictosidine glucosidase; THAS, tetrahydroalstonine synthase; GS, geissoschizine synthase; GO, geissoschizine oxidase, T16H, tabersonine 16-hydroxylase; 16OMT, tabersonine 16-O-methyltransferase; T3O, tabersonine 3-oxidase, T3R, tabersonine 3-reductase; NMT, tabersonine N-methyltransferase; D4H, desacetoxyvindoline 4-hydroxylase; DAT, deacetylvindoline O-acetyltransferase.

A.

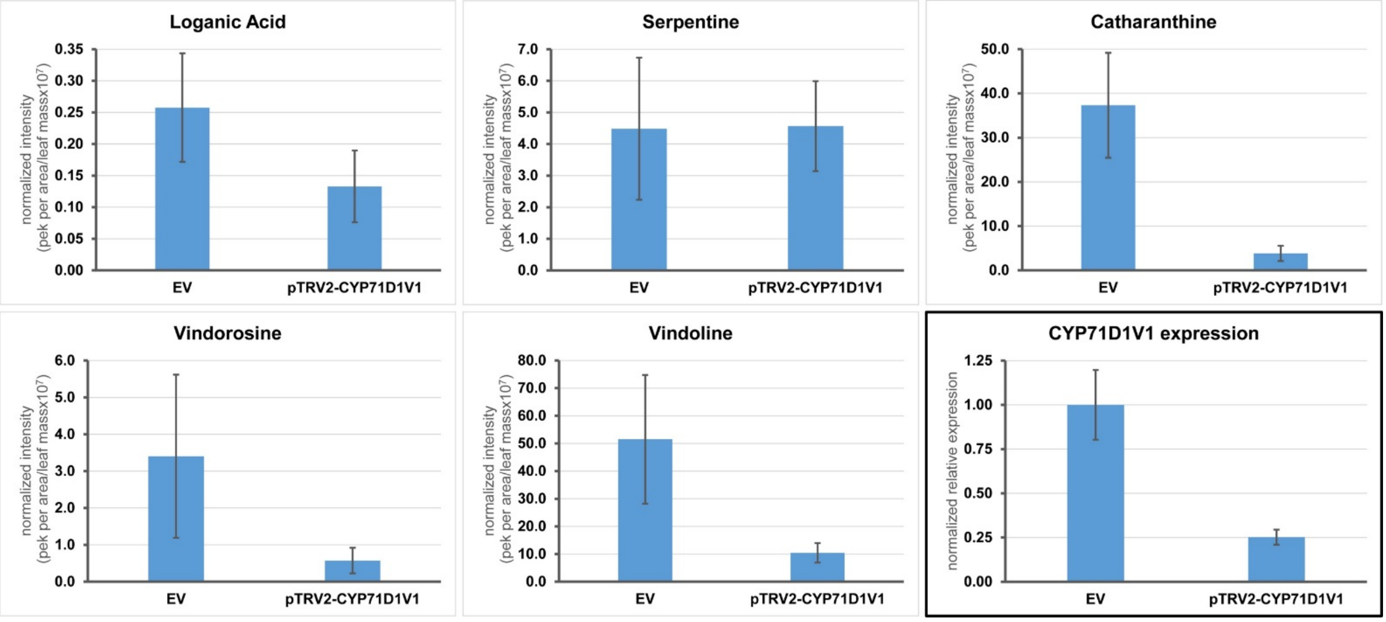

B.

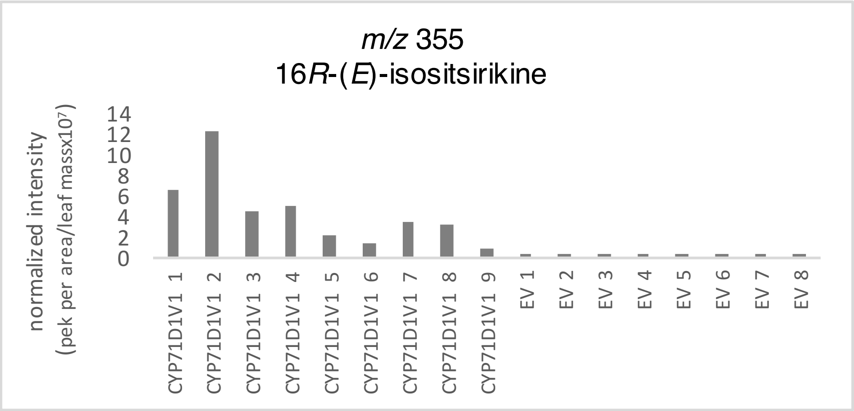

Supplementary Figure 2. Virus induced gene silencing (VIGS) of CYP71D1V1 (GO). A. Representative data of non-targeted metabolite analysis and qRT-PCR data in *C. roseus* silenced tissues. Alkaloids vindorosine, vindoline and catharanthine exhibit significant changes in *C. roseus* silenced tissues. Error bars in all cases represent the standard error of eight biological replicates. B. The levels of the compound *m/z* 355 (assigned as 16*R*-(*E*)-isositsirikine) in all replicates of silenced (CYP71D1V1) compared to empty vector (EV) negative controls.

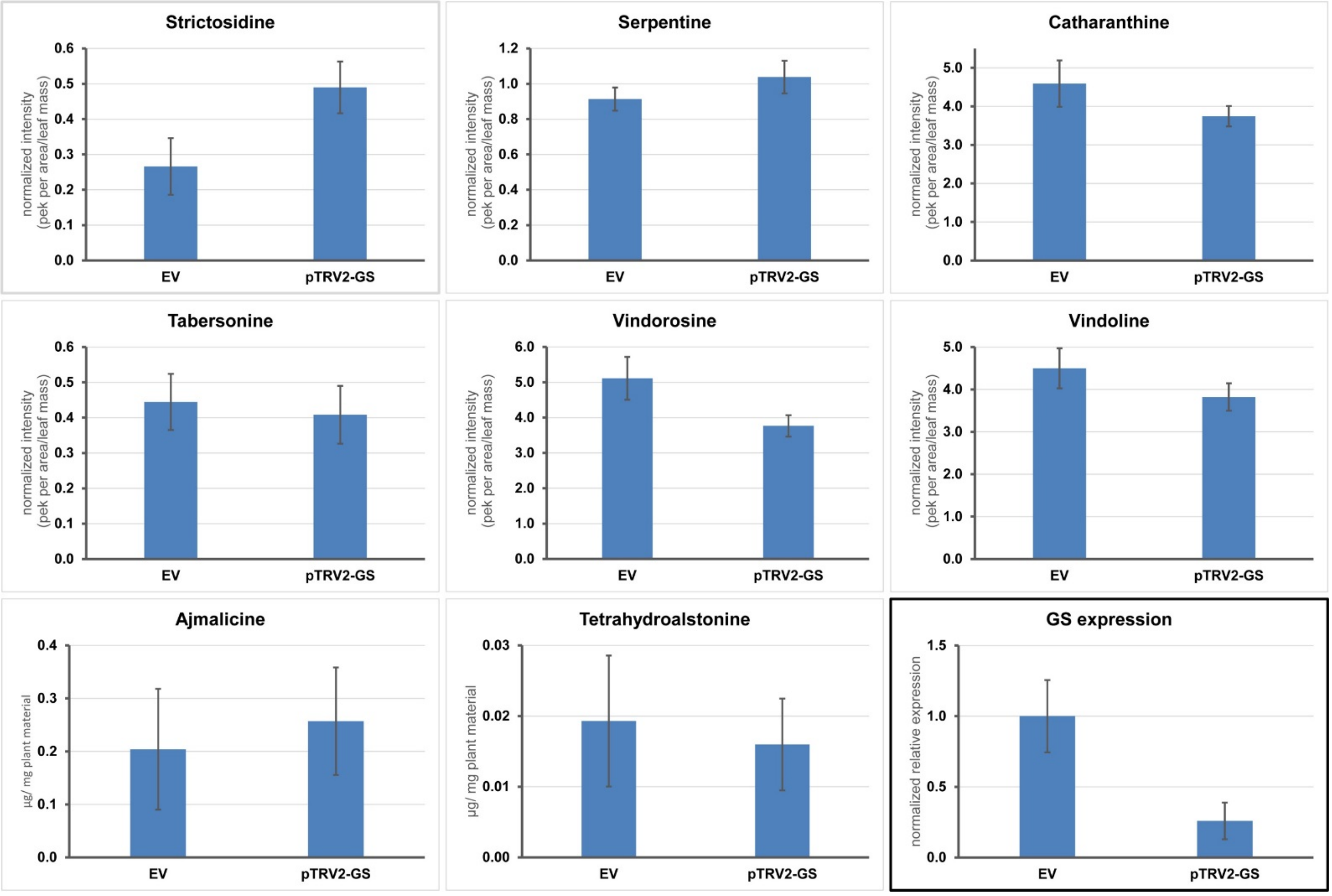

Supplementary Figure 3. VIGS of GS1. Representative data of non-targeted metabolite analysis, metabolite analysis of heteroyohimbines, ajmalicine and tetrahydroalstonine, and qRT-PCR data in *C. roseus* silenced tissues. Only strictosidine exhibits any significant changes. Error bars in all cases represent the standard error of eight biological replicates.

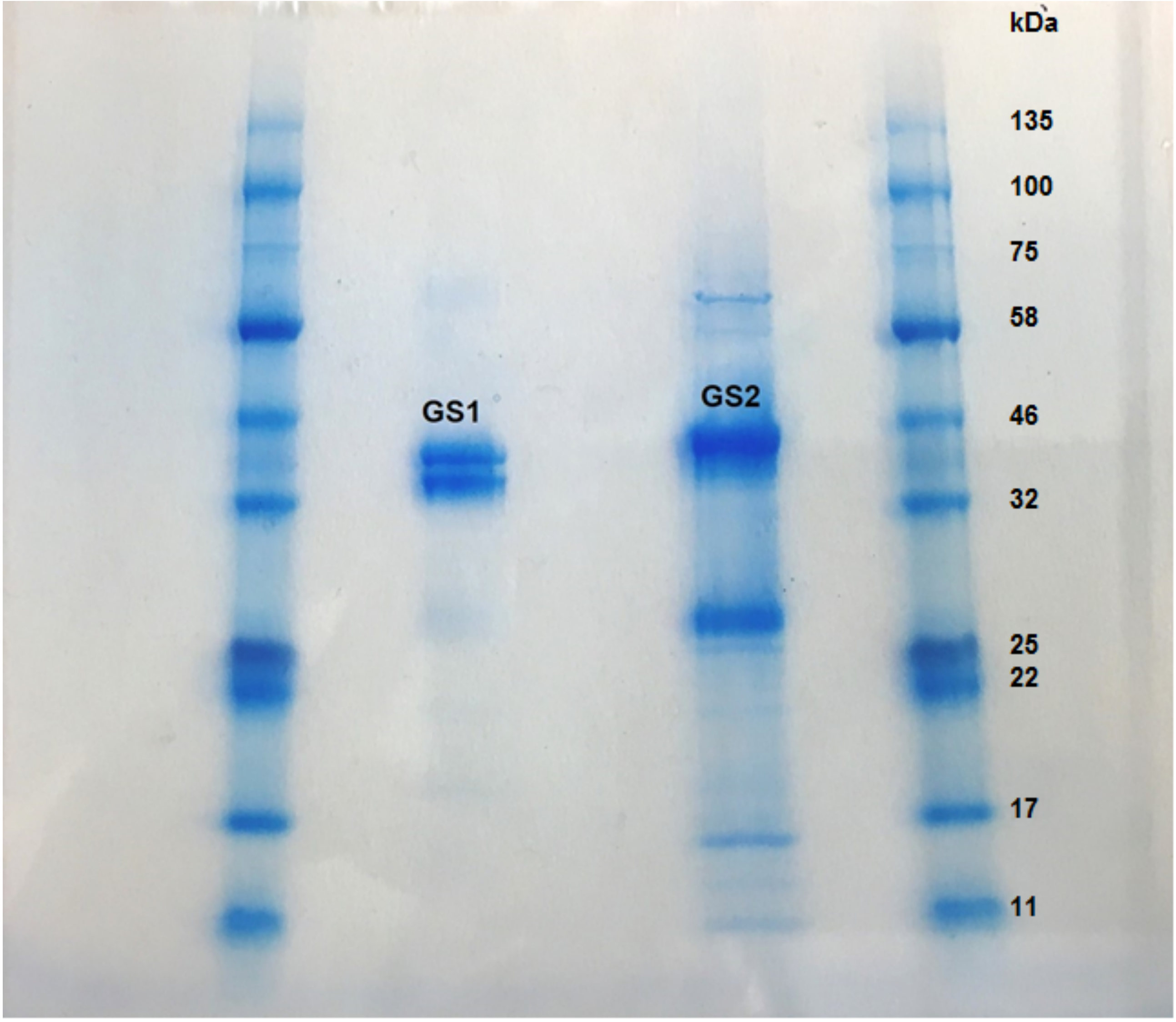

Supplementary Figure 4. SDS-PAGE of purified GS1 and GS2. Blue Prestained Protein Standard, Broad Range be New England Buffers was used as protein ladder.

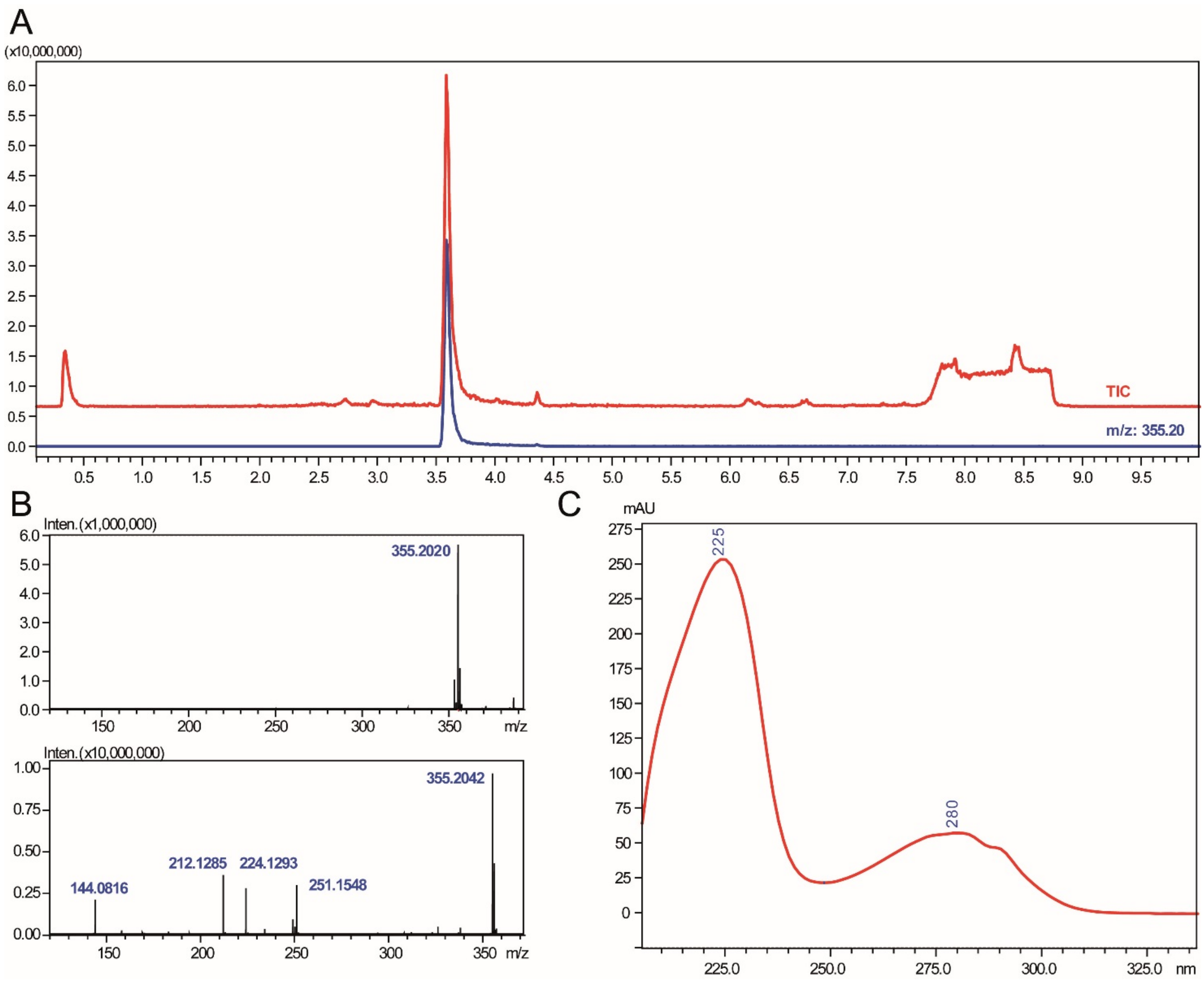

Supplementary Figure 5. Chromatographic (A), MS (B) and UV-Vis data (C) of isolated (16*R*)-E-isositsirikine (*1*).

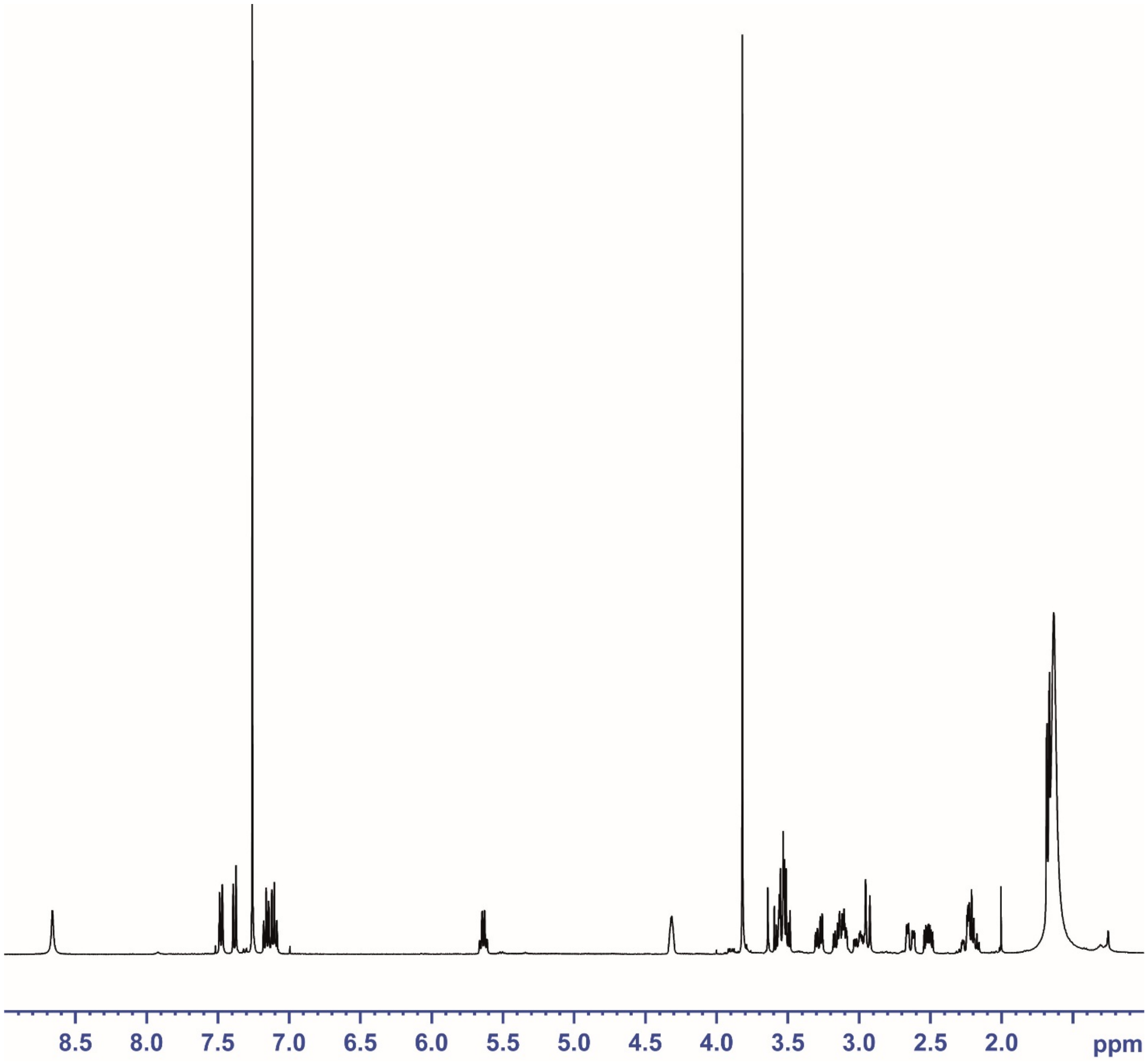

Supplementary Figure 6. ^{1}H-NMR spectrum of (16*R*)-E-isositsirikine (*1*).

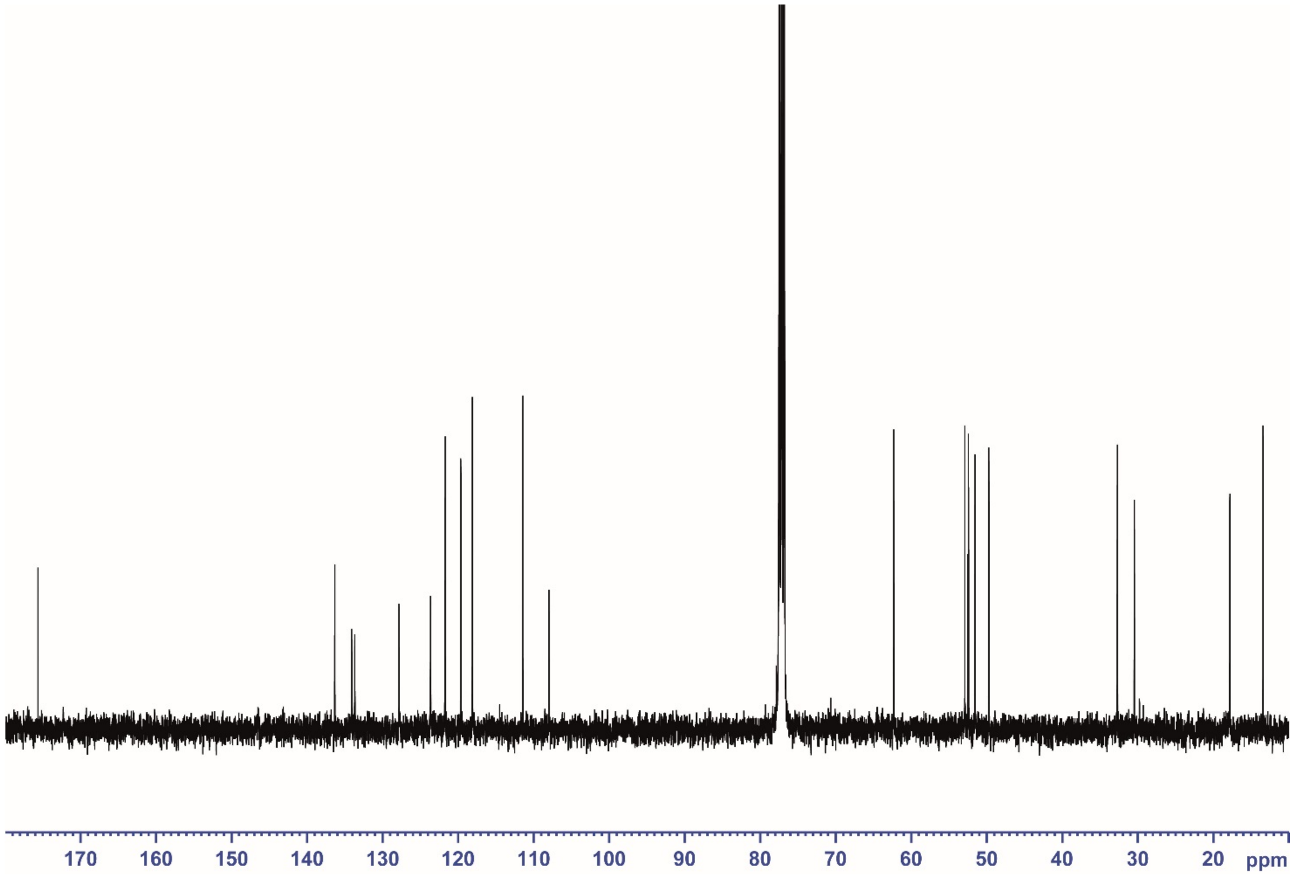

Supplementary Figure 7. ^{13}C-NMR spectrum of (16*R*)-E-isositsirikine (*1*).

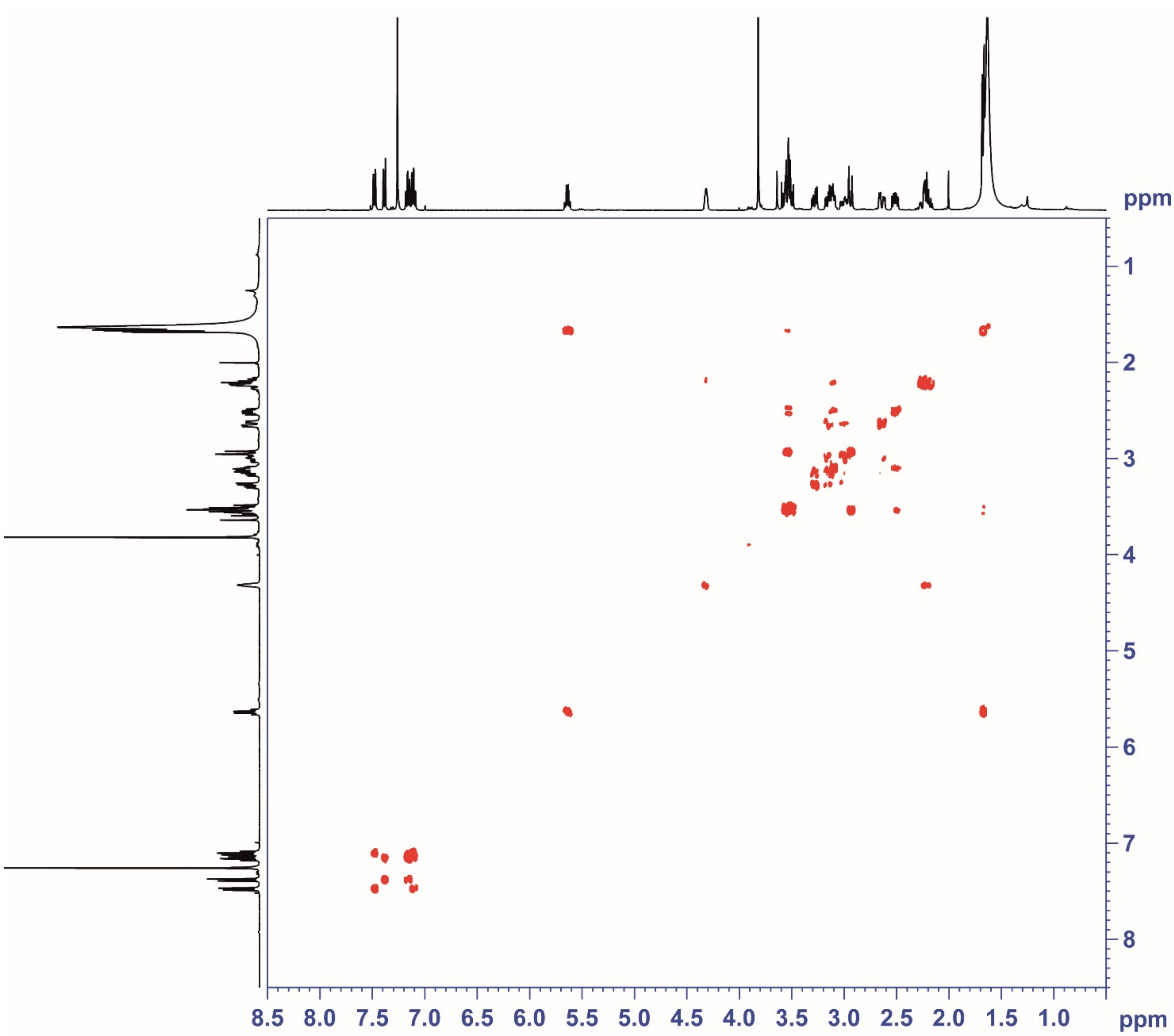

Supplementary Figure 8. ^{1}H ^{1}H-COSY spectrum of (16*R*)-E-isositsirikine (*1*).

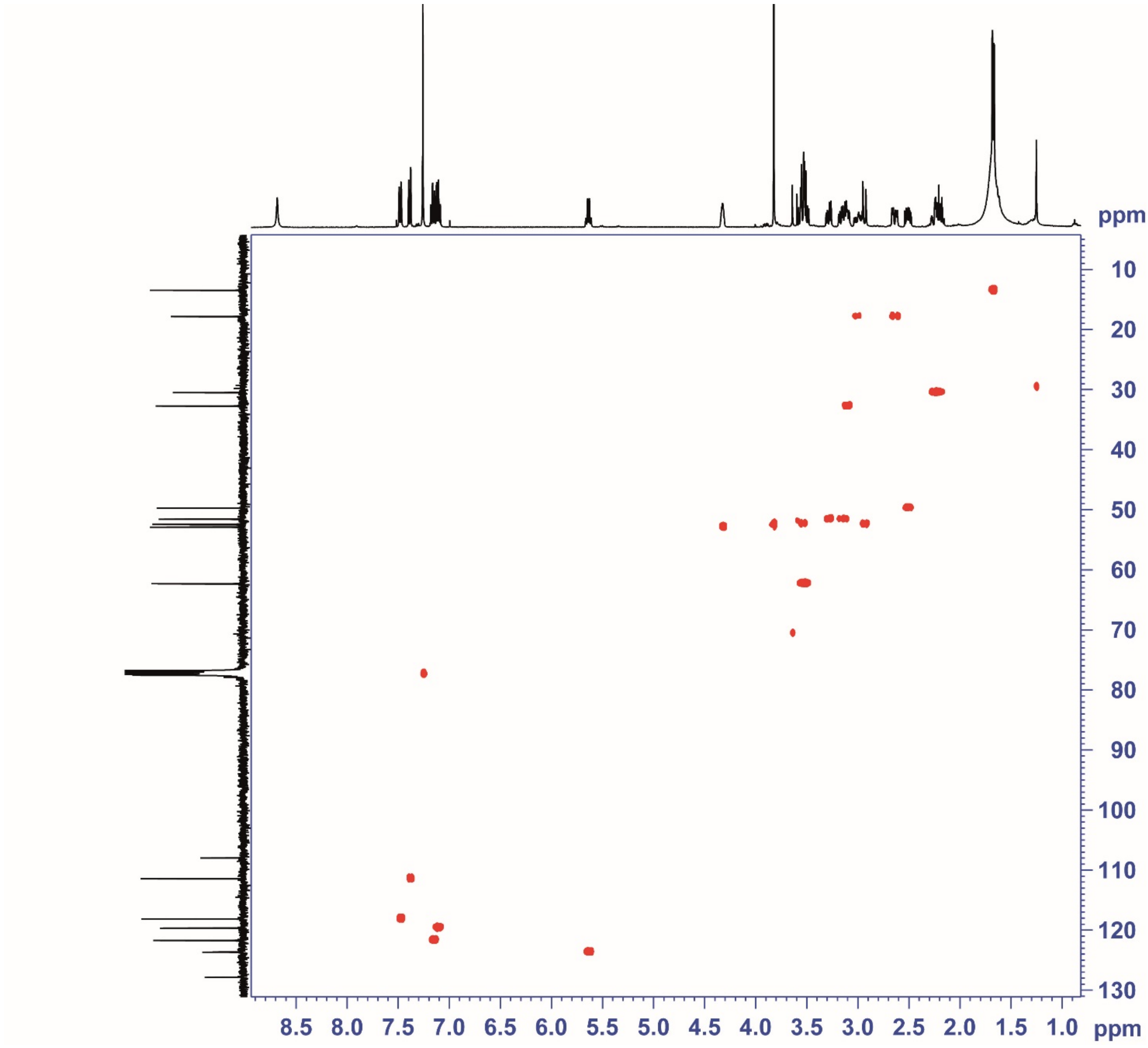

Supplementary Figure 9. ^{1}H ^{13}C-HSQC spectrum of (16*R*)-E-isositsirikine (*1*).

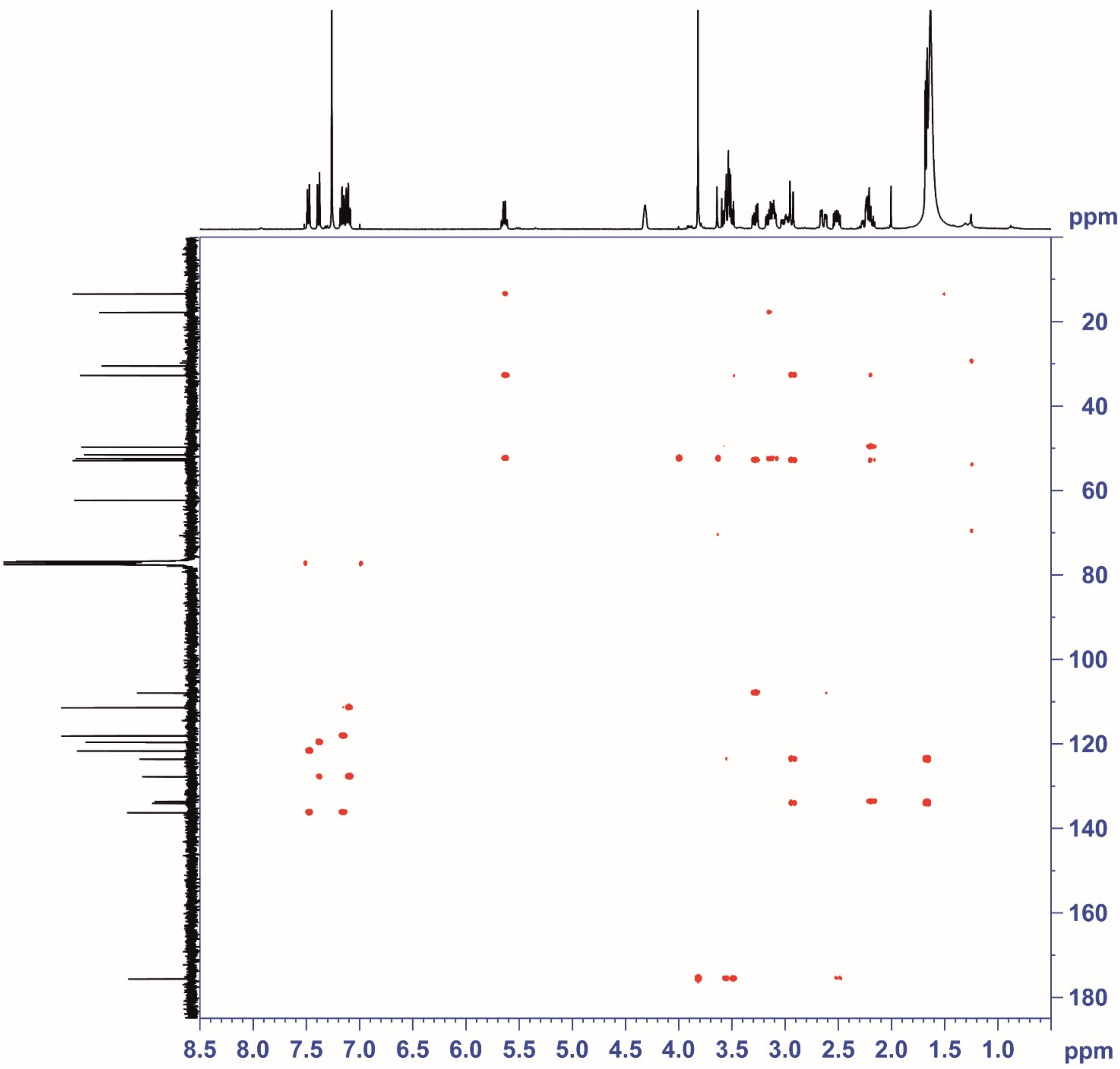

Supplementary Figure 10. ^{1}H ^{13}C-HMBC spectrum of (16*R*)-E-isositsirikine (*1*).

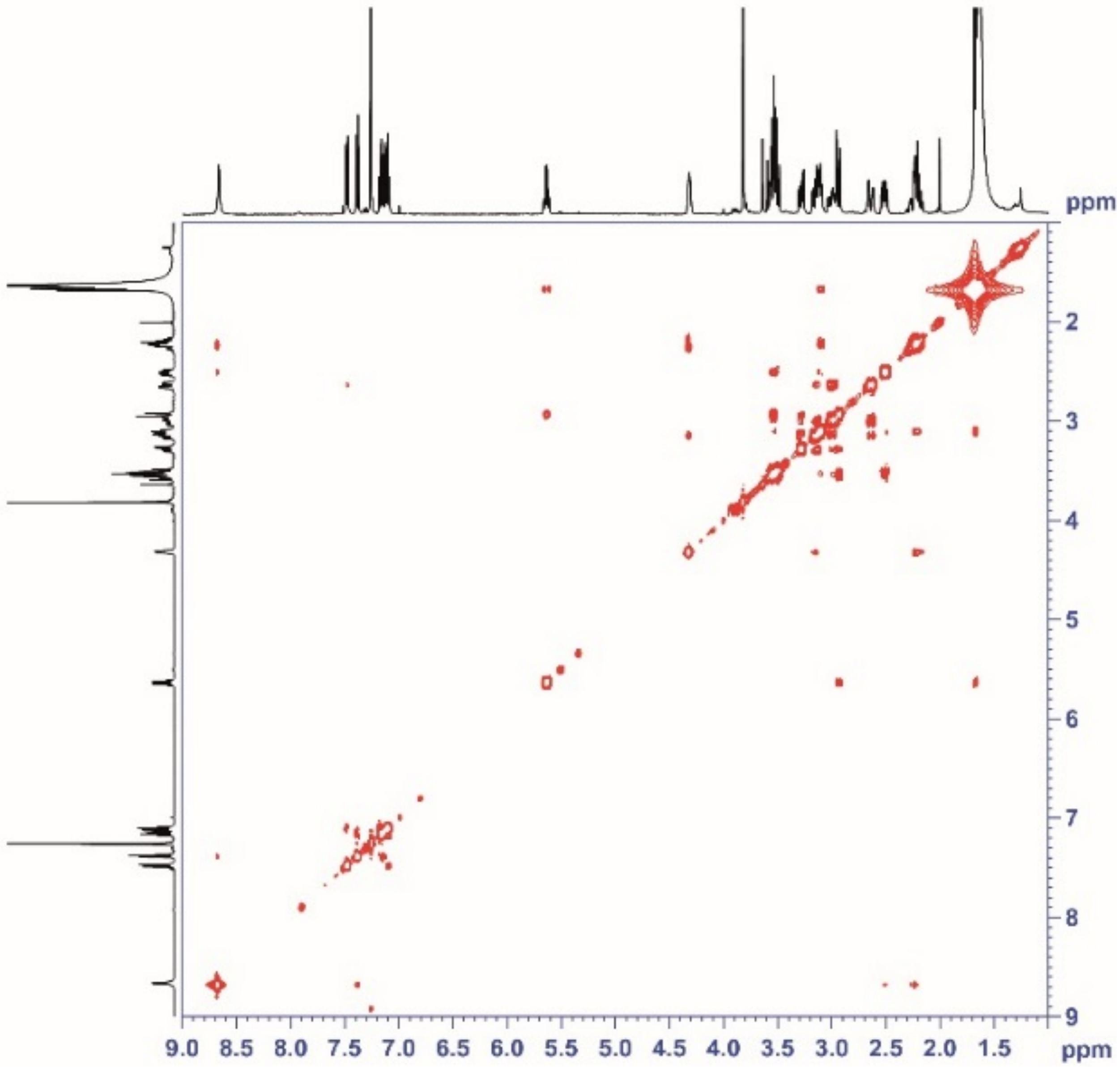

Supplementary Figure 11. ROESY spectrum of (16*R*)-E-isositsirikine (*1*).

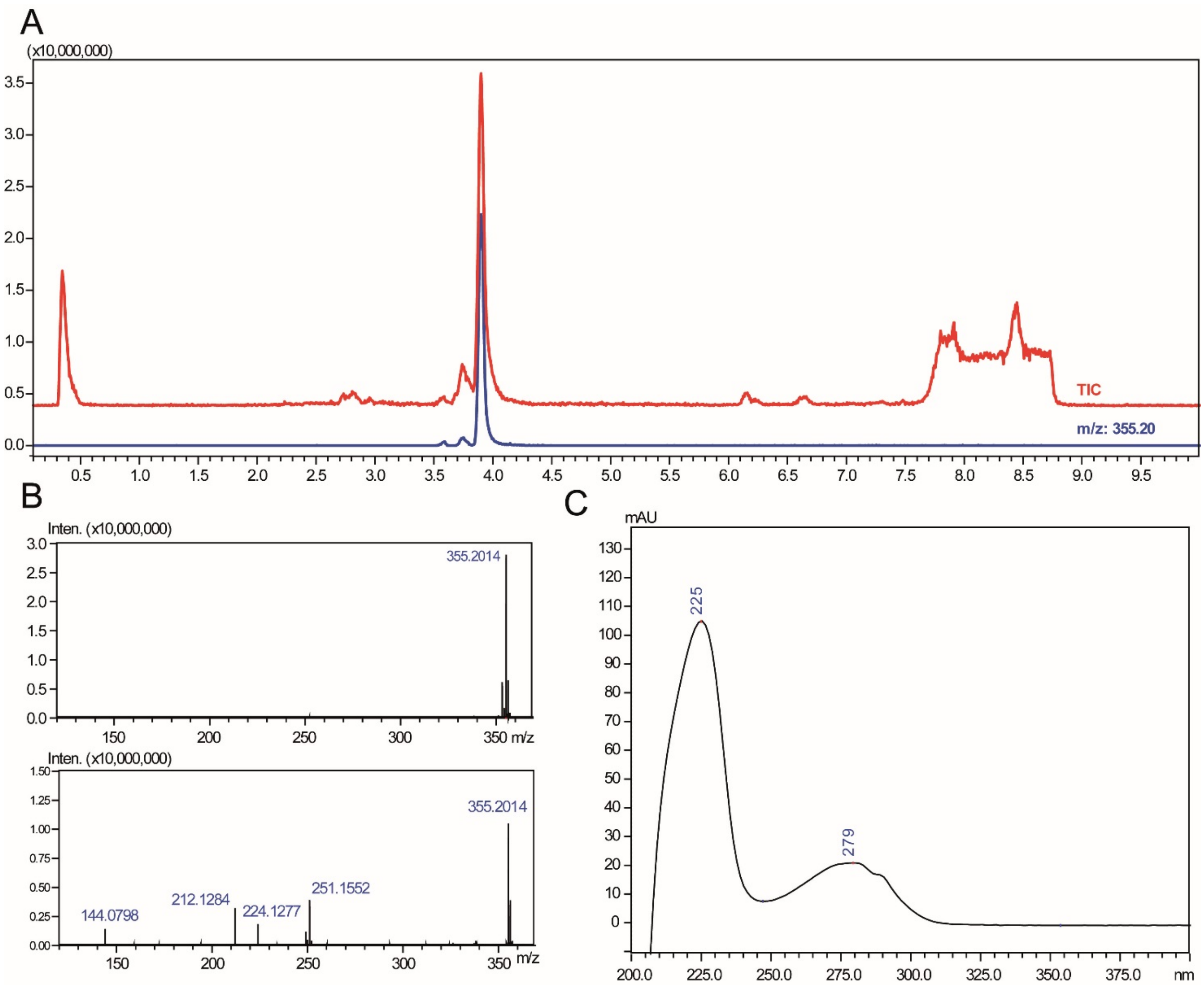

Supplementary Figure 12. Chromatographic (A), MS (B) and UV-Vis data (C) of isolated (16*R*)-Z-isositsirikine (*2*).

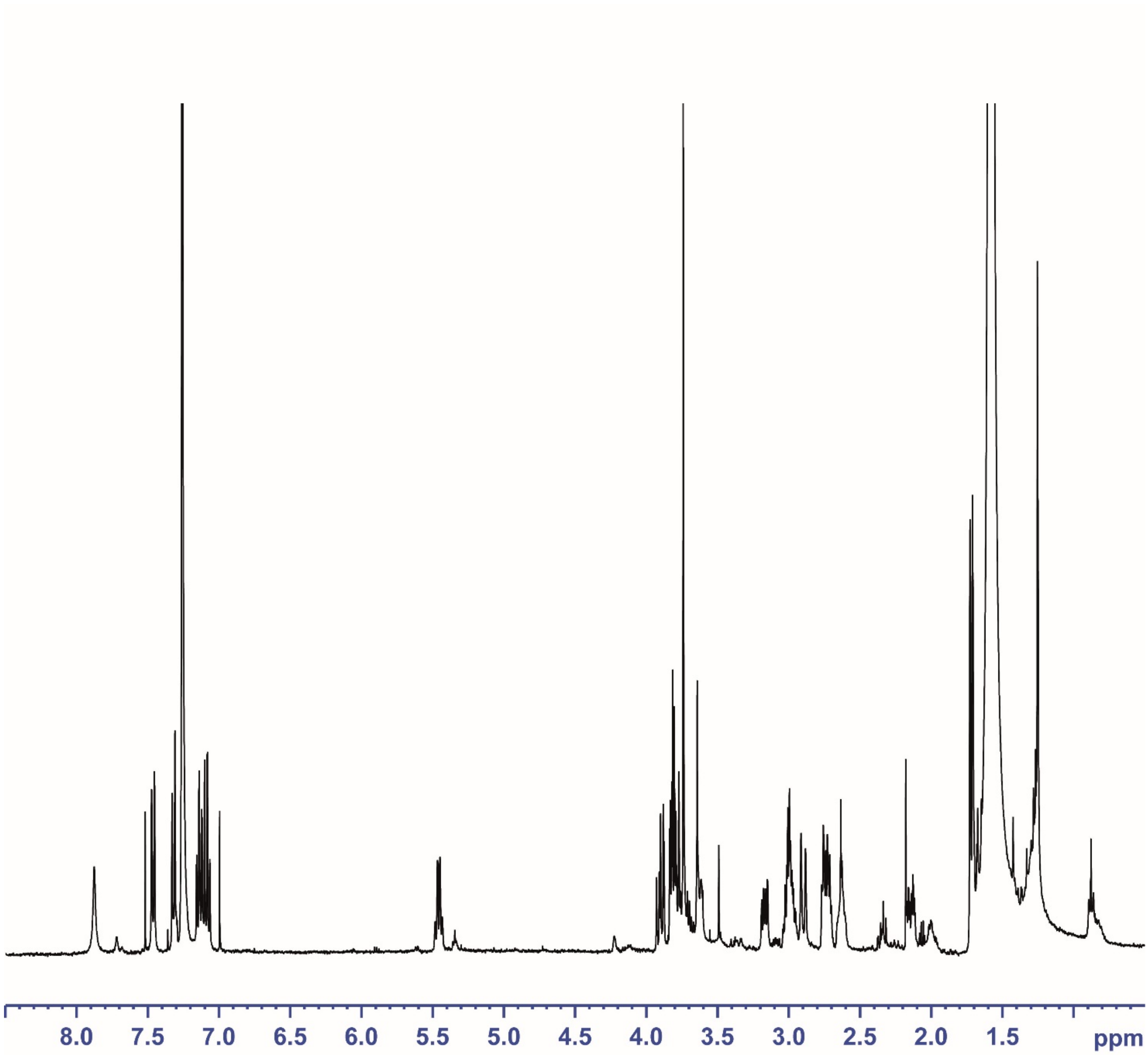

Supplementary Figure 13. ^{1}H-NMR spectrum of (16*R*)-Z-isositsirikine (*2*).

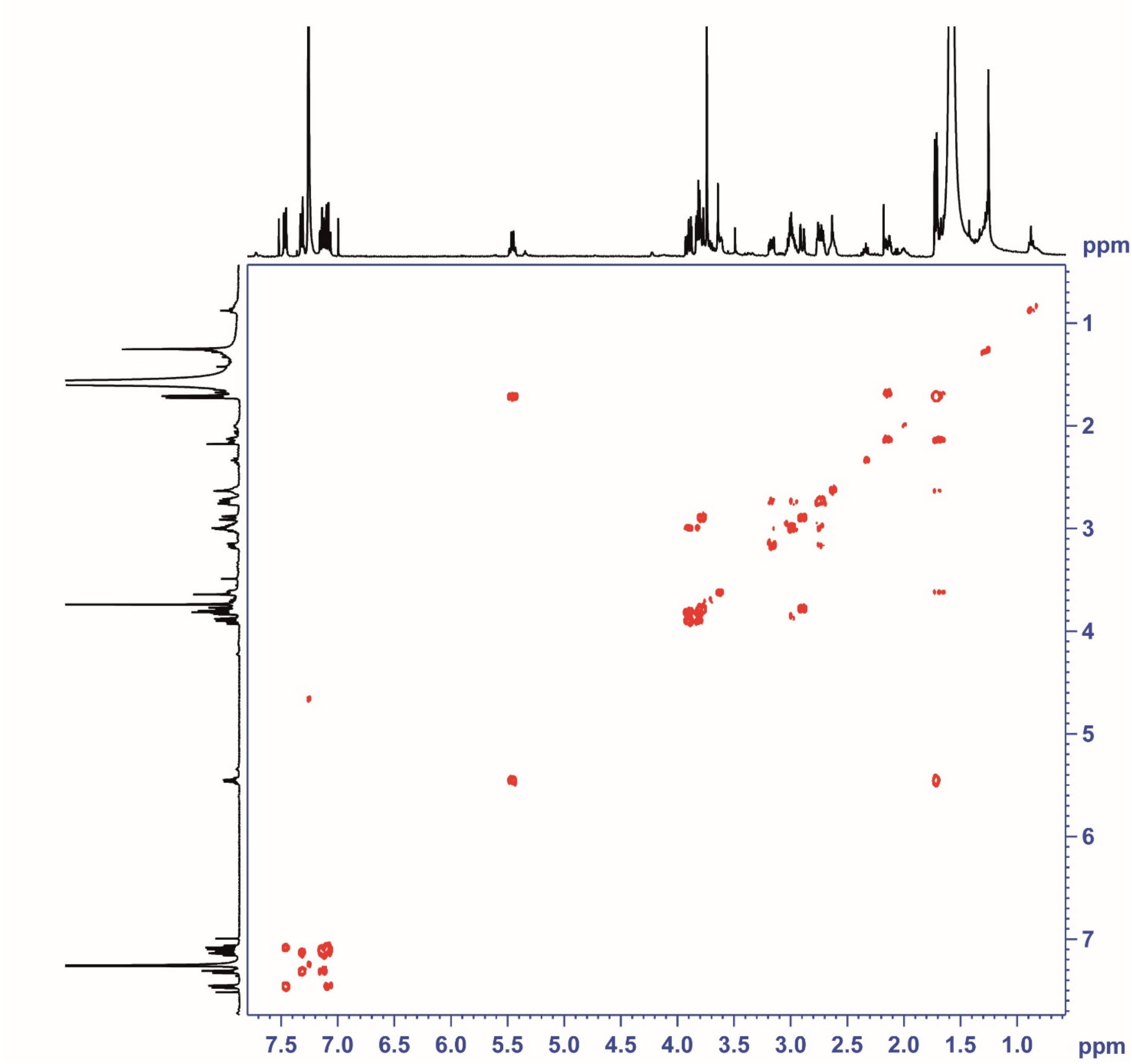

Supplementary Figure 14. ^{1}H ^{1}H-COSY spectrum of (16*R*)-Z-isositsirikine (*2*).

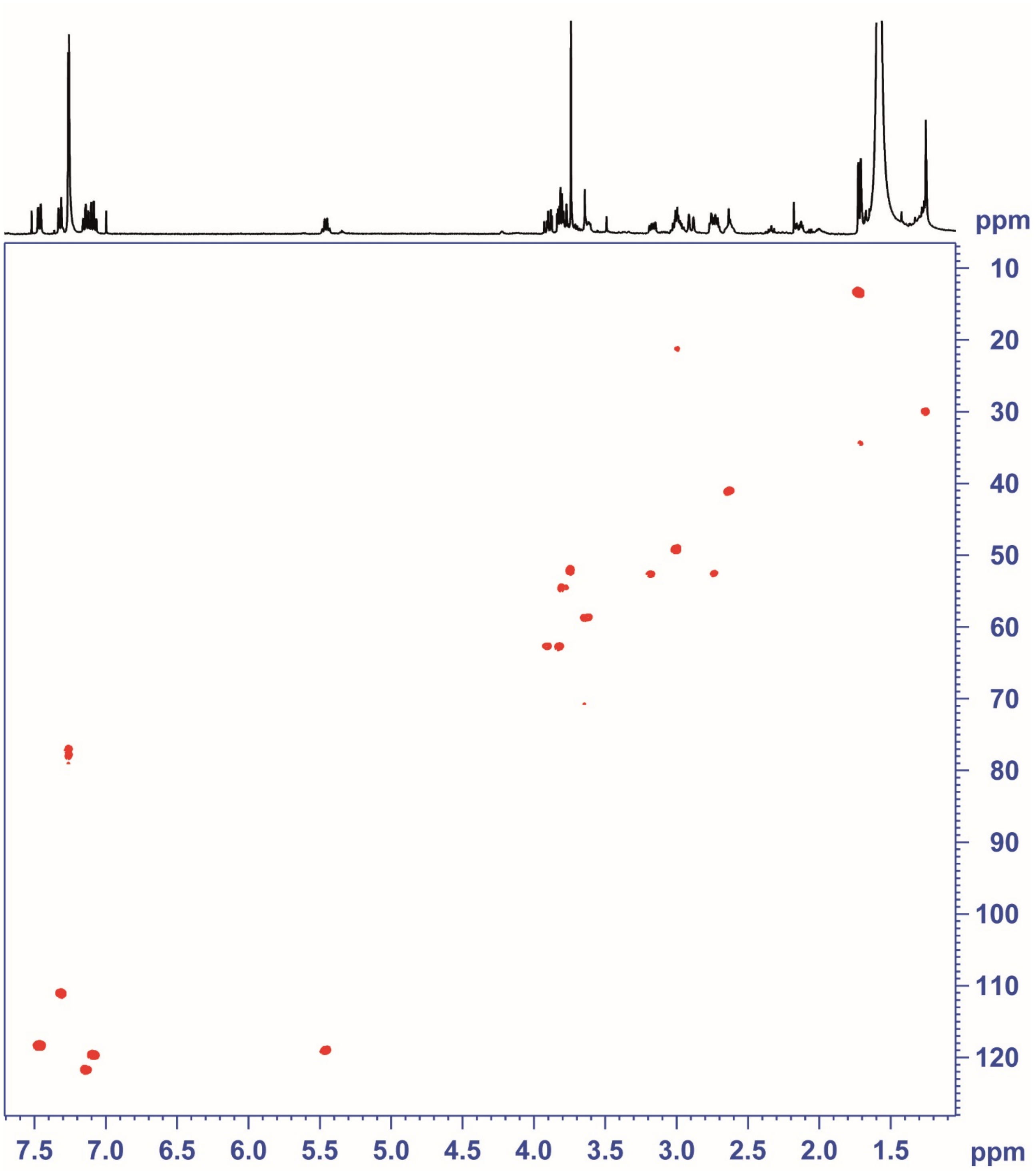

Supplementary Figure 15. ^{1}H ^{13}C-HSQC spectrum of (16*R*)-Z-isositsirikine (*2*).

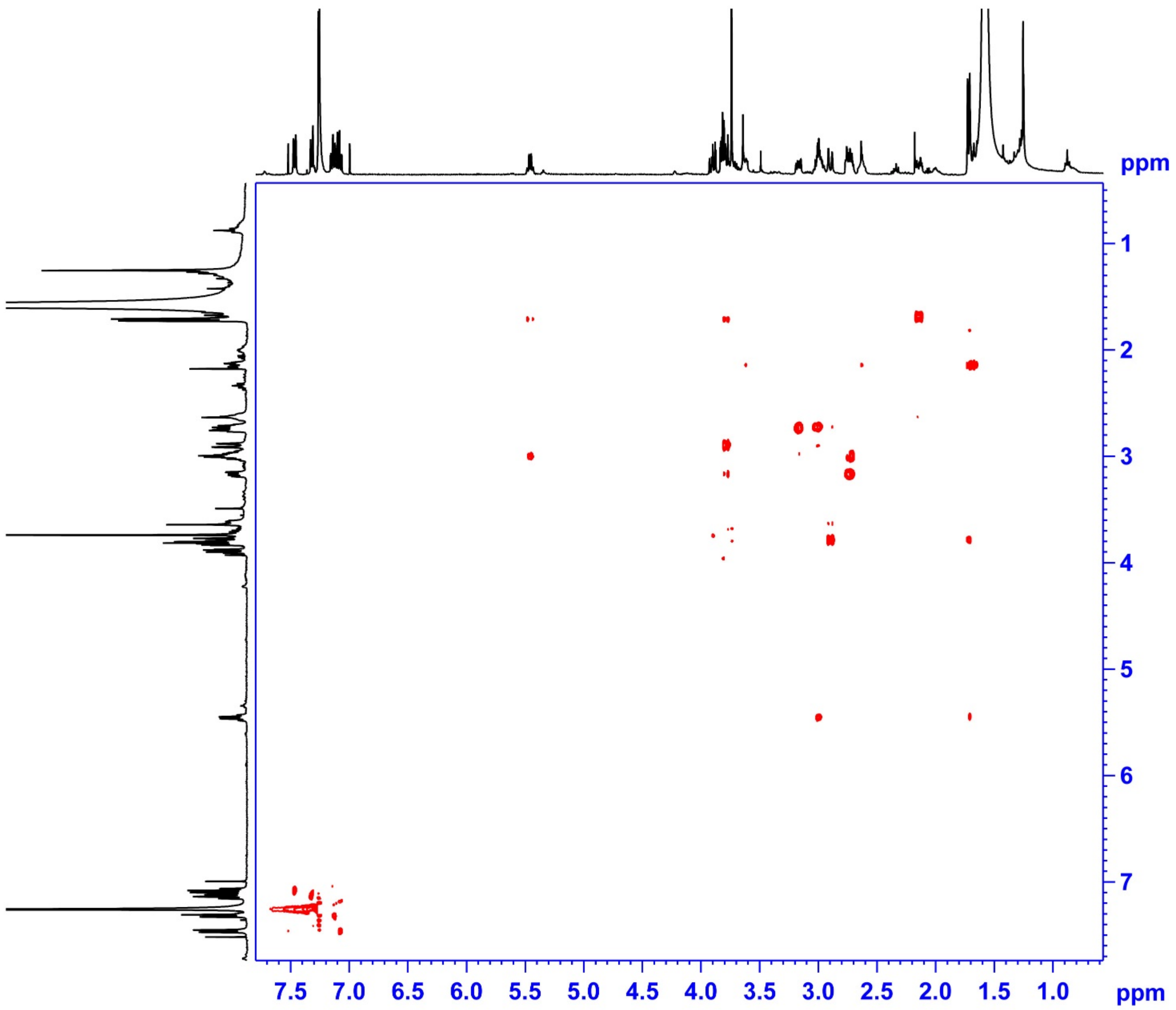

Supplementary Figure 16. ROESY spectrum of (16*R*)-Z-isositsirikine (*2*).

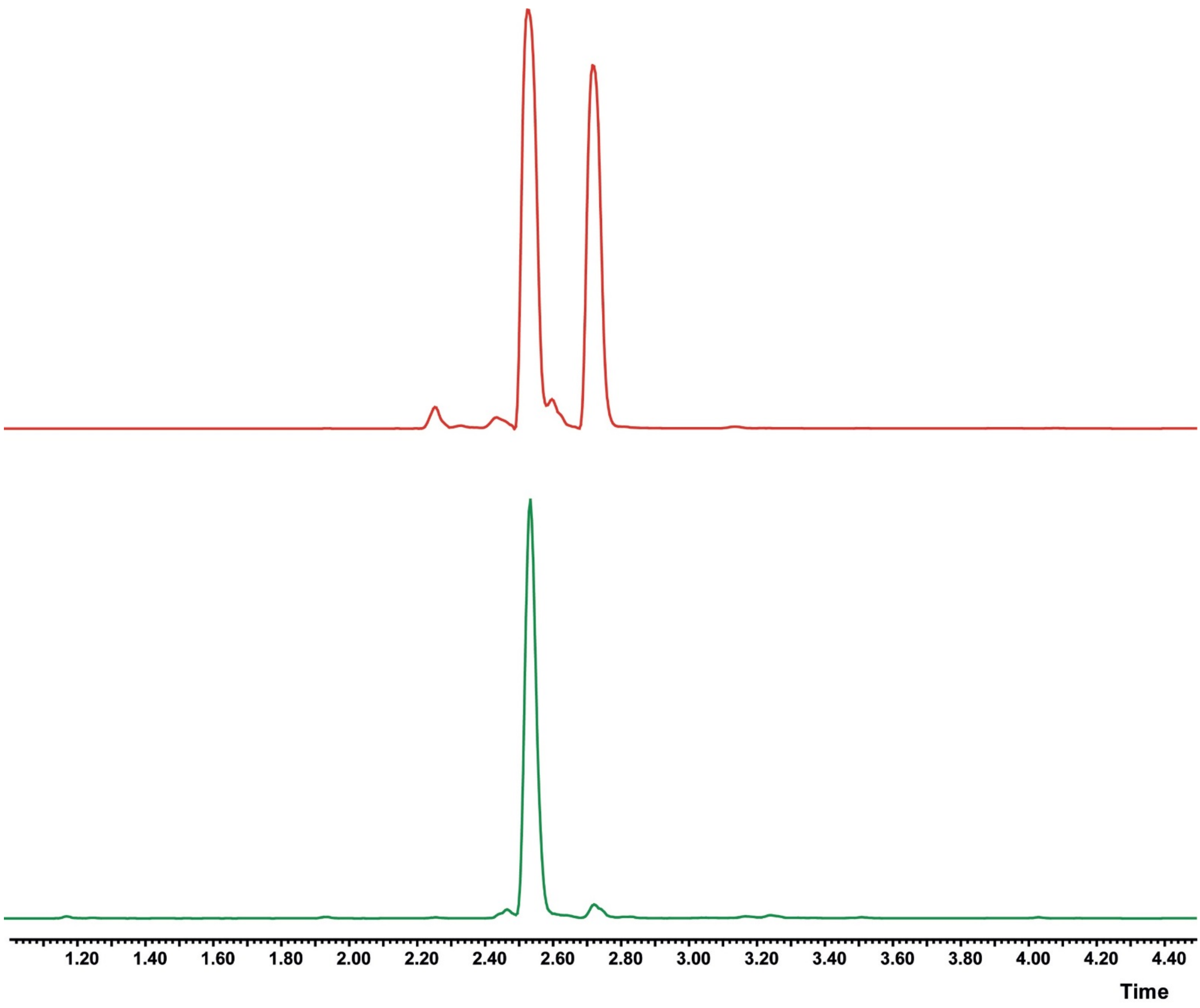

Supplementary Figure 17. LC-MS chromatograms (MRM at *m/z* 355 for isositsirikine) of GS1 enzyme assay with strictosidine aglycone (in red) compared to a chromatogram of virus induced gene silenced GO (CYP71D1V1) *C. roseus* plant tissue extract (green). While the GS1 (and GS2) produce 16*R*-*E*-isositsirikine and 16*R*-*Z*-isositsirikine, the silencing of GO (CYP71D1V1) in *C. roseus* seedling results to the accumulation of 16*R*-*E*-isositsirikine.

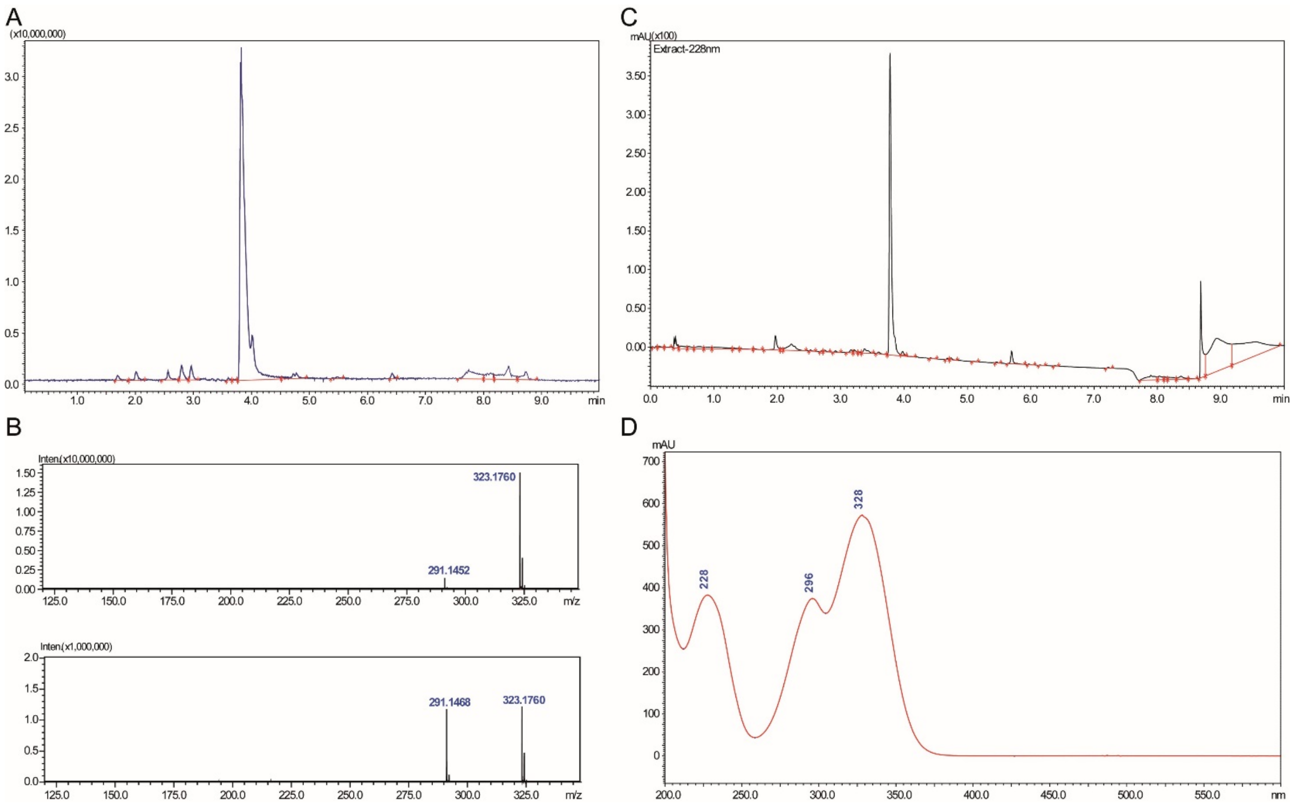

Supplementary Figure 18. Chromatographic (A), MS (B) and UV-Vis data (C) of isolated akuammicine (*3*).

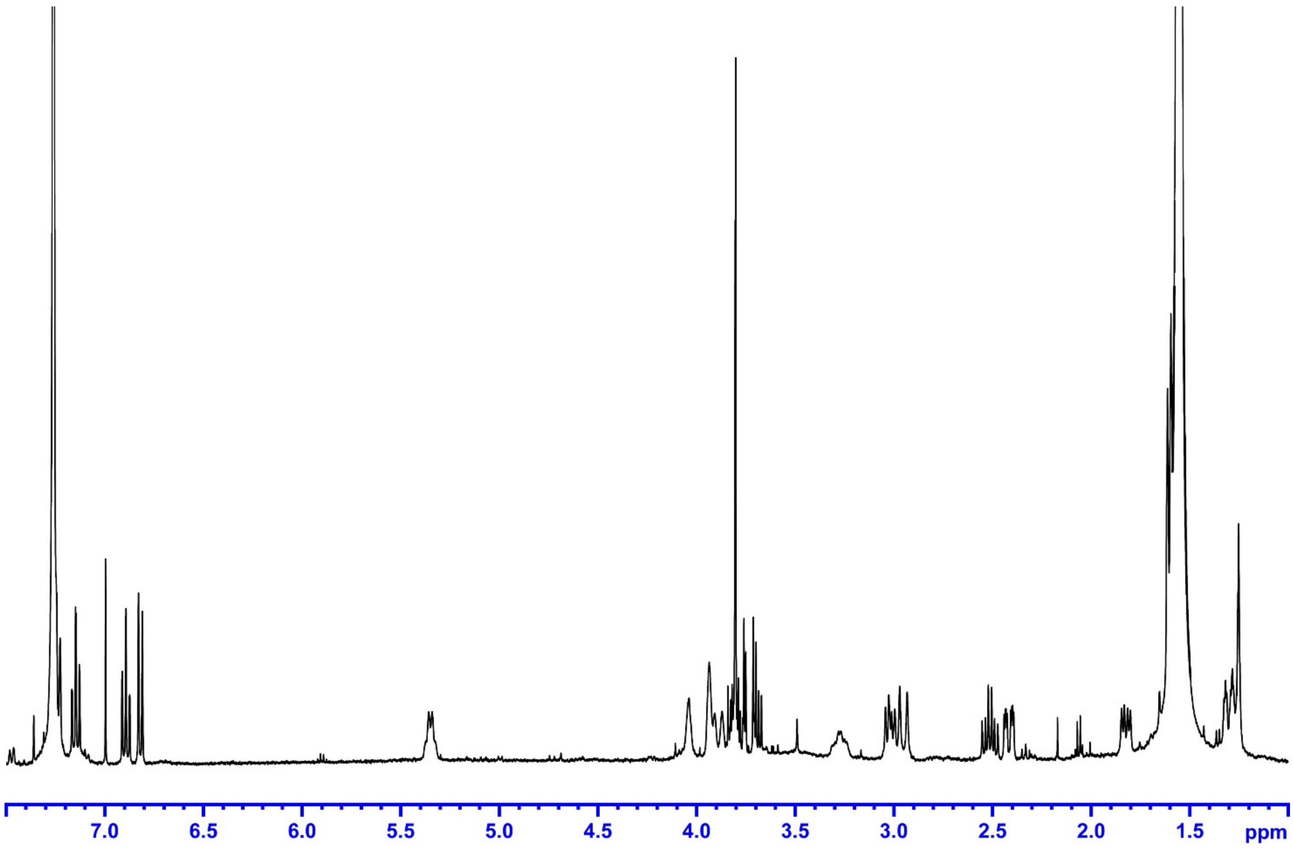

Supplementary Figure 19. ^{1}H-NMR spectrum of akuammicine (*3*).

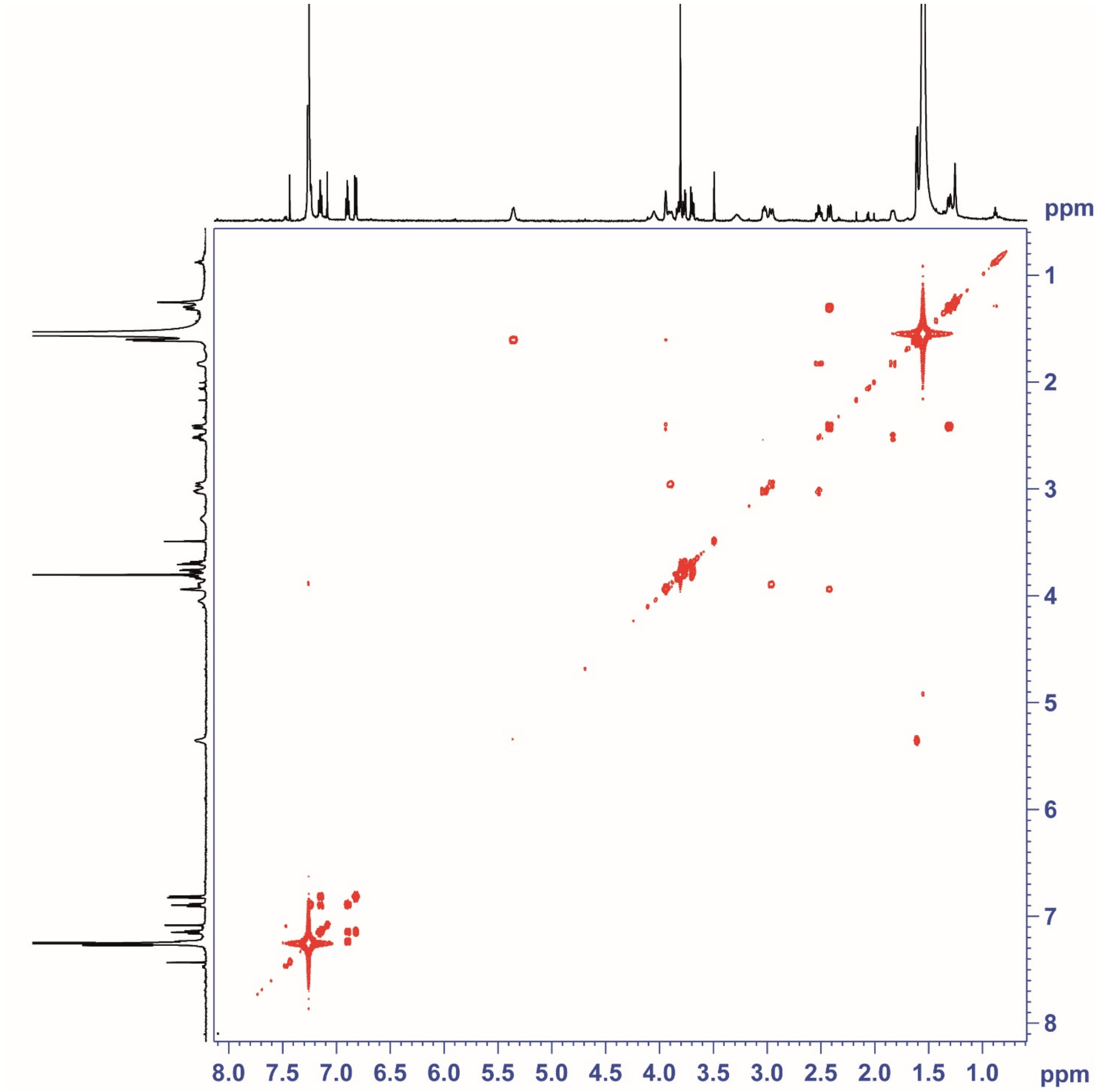

Supplementary Figure 20. ^{1}H ^{1}H-COSY spectrum of akuammicine (*3*).

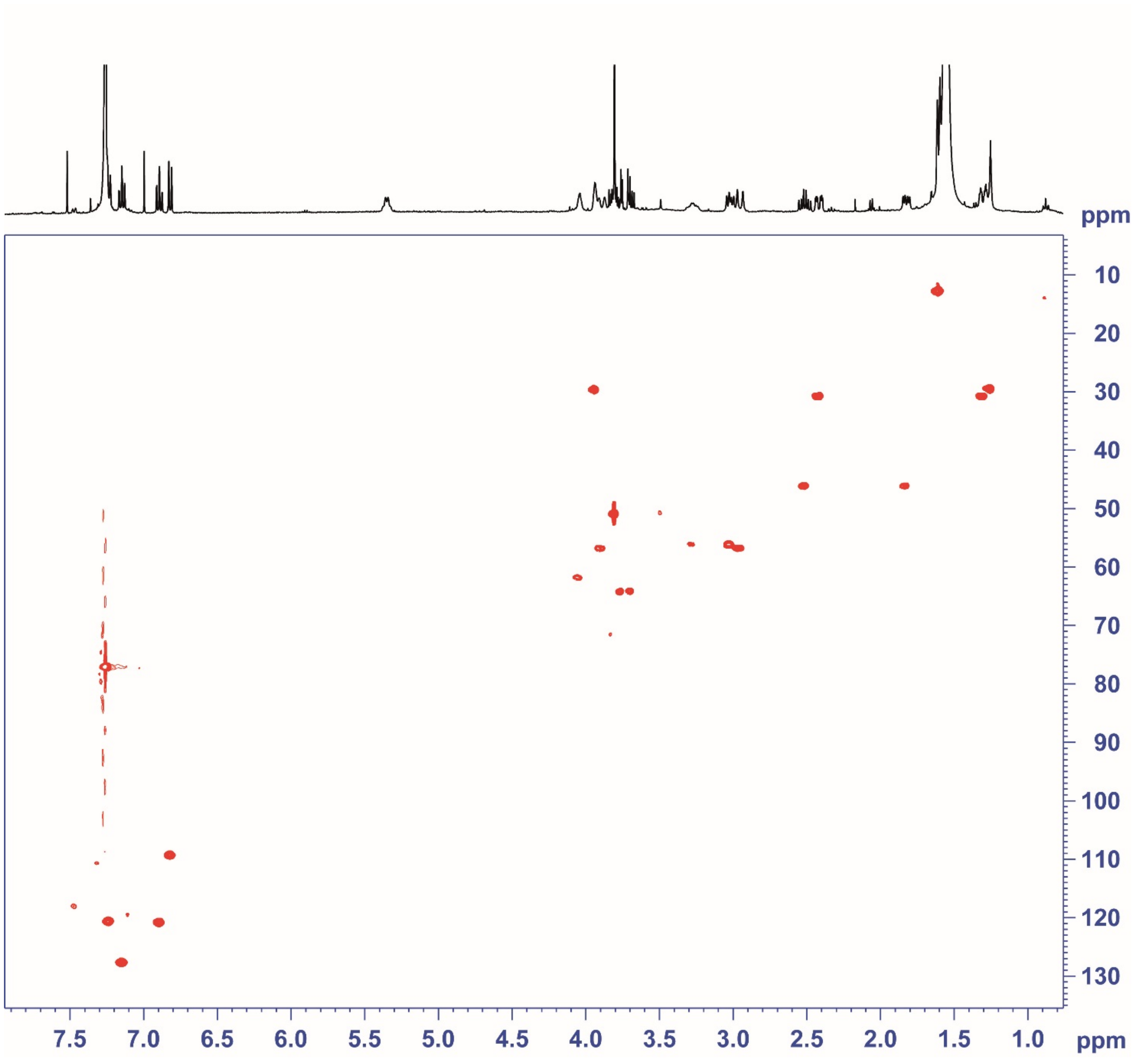

Supplementary Figure 21. ^{1}H ^{13}C-HSQC spectrum of akuammicine (*3*).

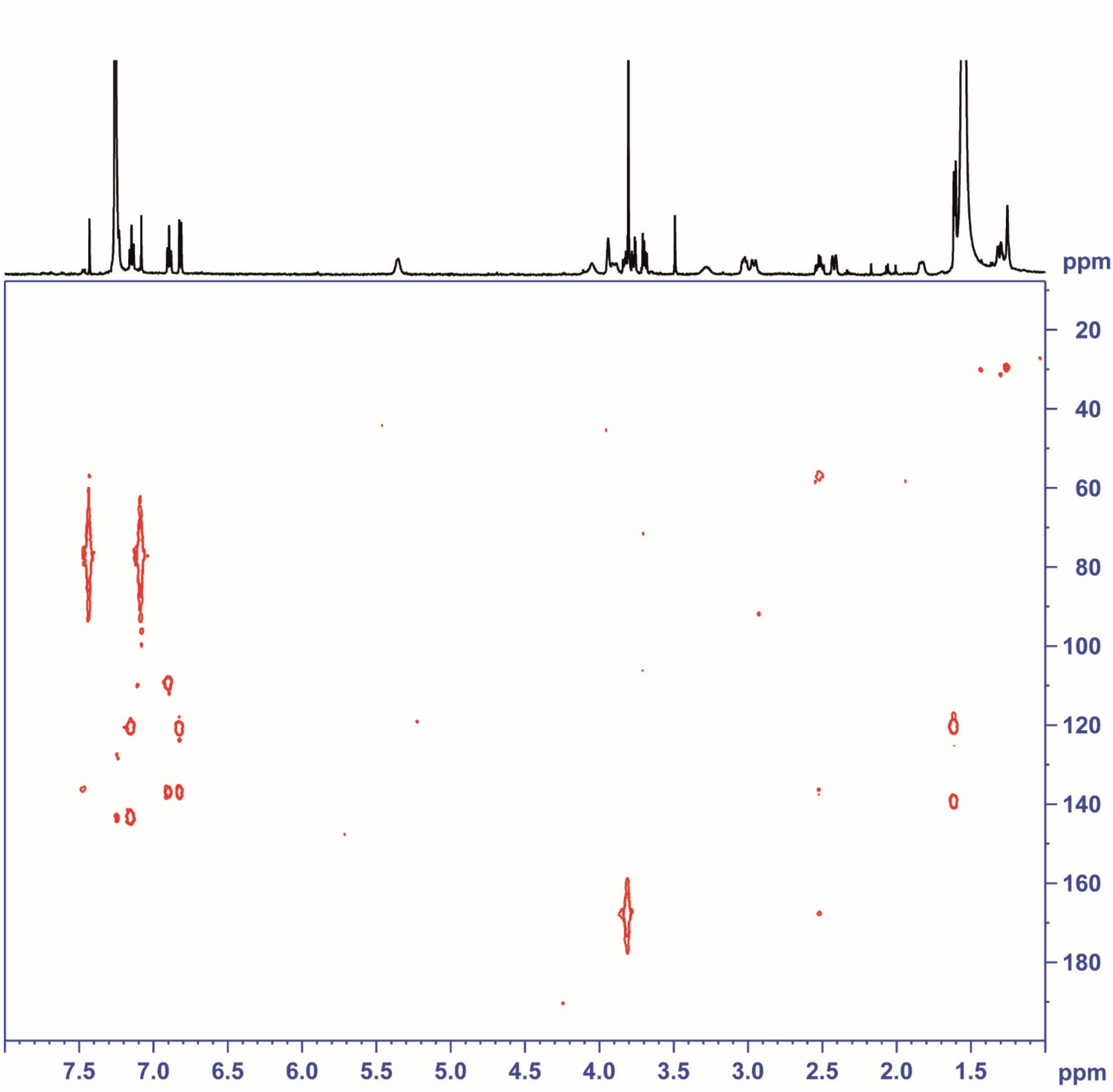

Supplementary Figure 22. ^{1}H ^{13}C-HMBC spectrum of akuammicine (*3*).

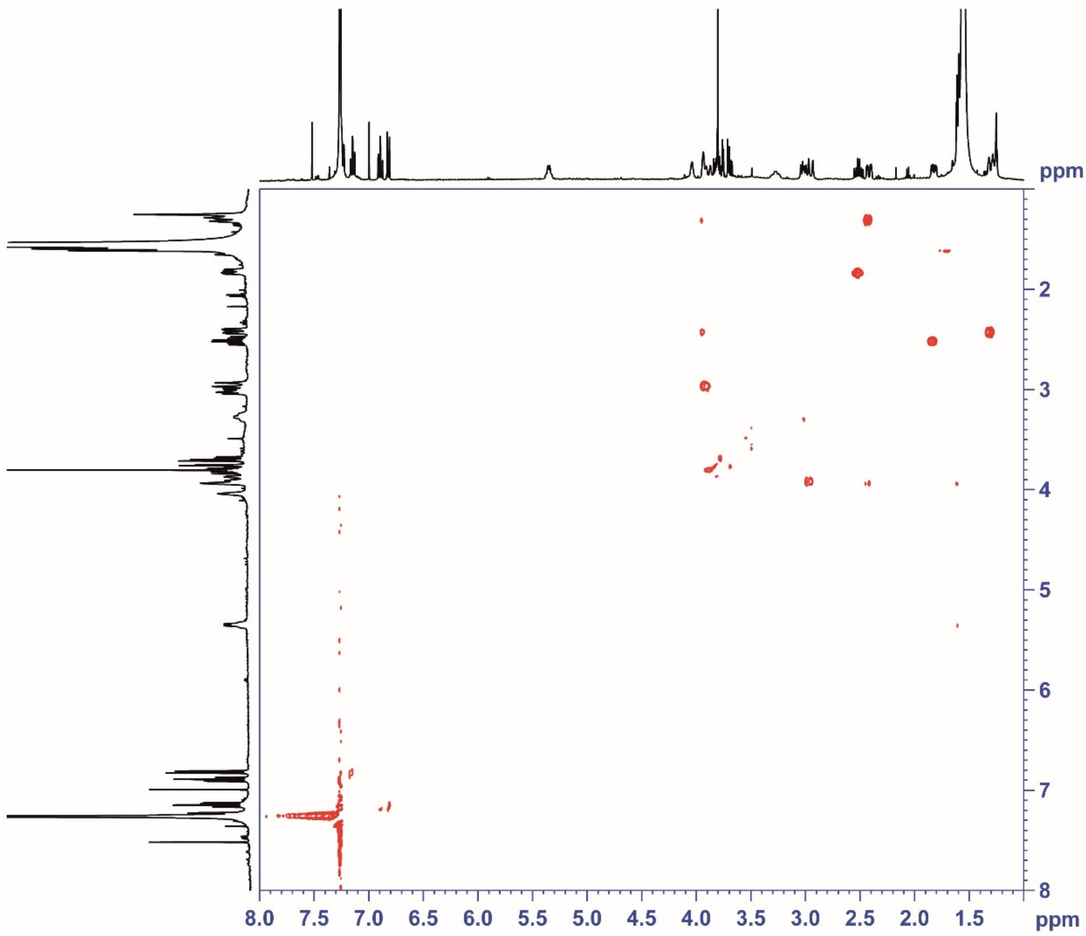

Supplementary Figure 23. ROESY spectrum of akuammicine (*3*).

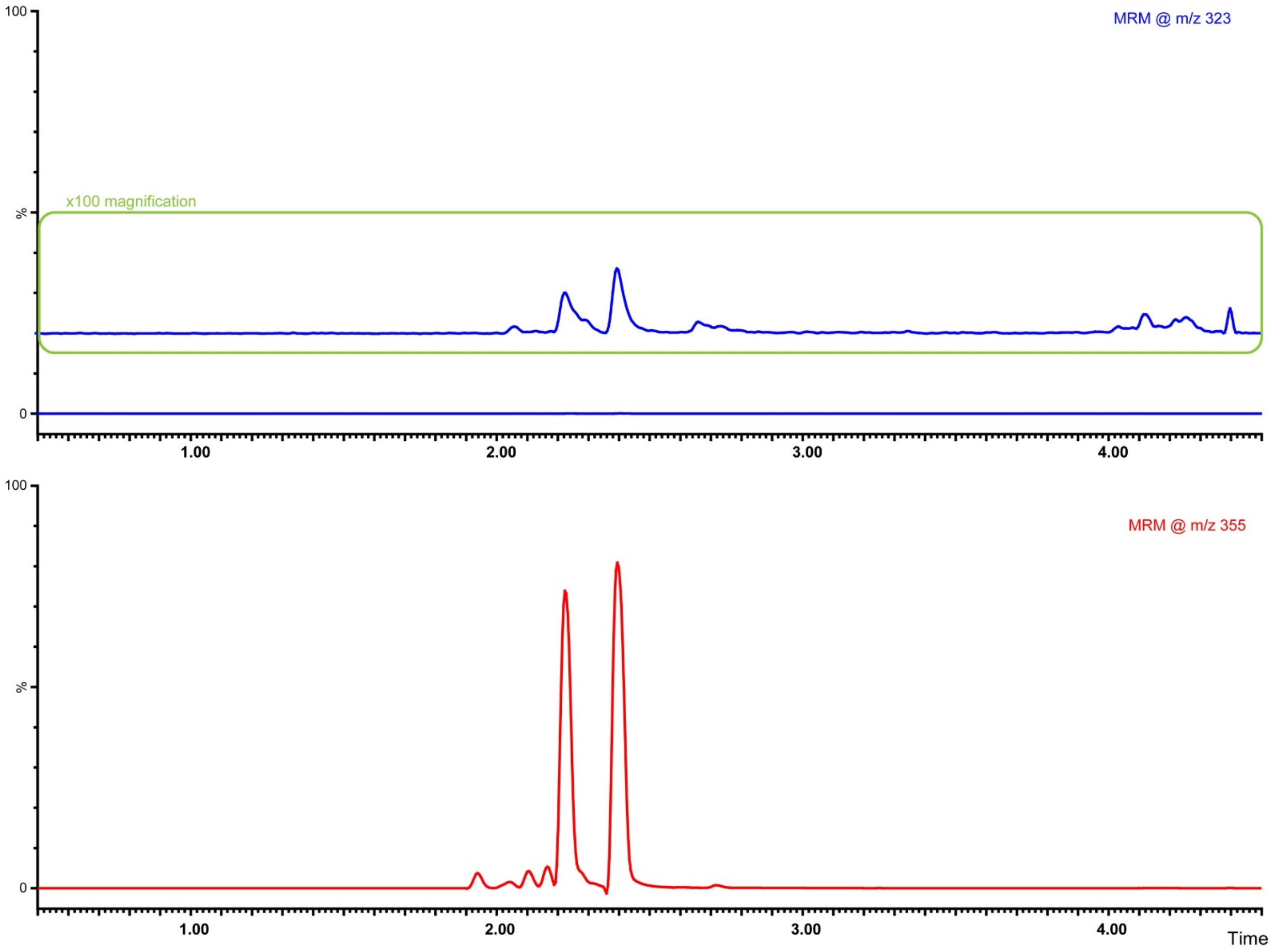

Supplementary Figure 24. LC-MS chromatograms of GS1 enzyme assay with strictosidine aglycone with later addition (30 min after addition of GS1 to strictosidine aglycone) of GO (CYP71D1V1). The formation of akuammicine is observed only when GS1 and GO (CYP71D1V1) are added at the same time to enzyme assay. In blue is shown the MRM at m/z 323 for akuammicine (with additional 100 times magnification in green frame) and in red is shown the MRM at m/z 355 for isositsirikines.

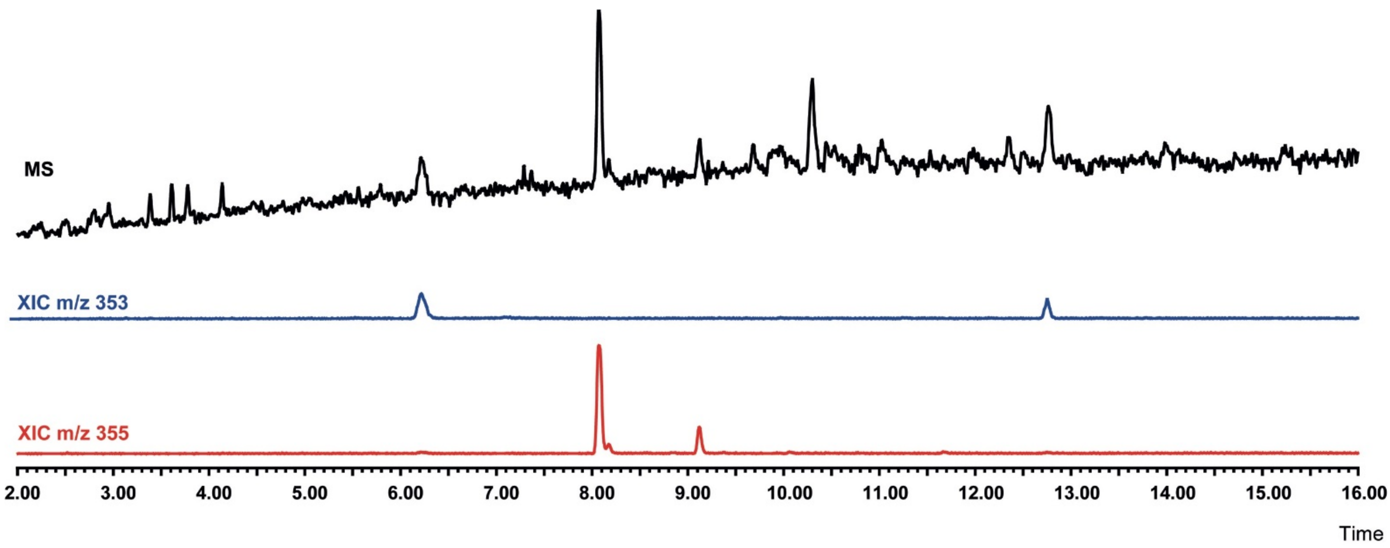

Supplementary Figure 25. LC-MS chromatogram of GS1 enzyme assay. The Total Ion Chromatogram (TIC) is depicted in black, the extracted ion chromatogram (XIC) at *m/z* 353 is depicted in blue, and in red is the extracted ion chromatogram (XIC) at *m/z* 355. The compounds with *m/z* 355 eluting at 8.1 min and 9.1 min are (16*R*)-*E*-isositsirikine and (16*R*)-*Z*-isositsirikine, respectively. The compound with *m/z* 353 eluting at 6.3 min could not be isolated, but the mass is consistent with geissoschizine.

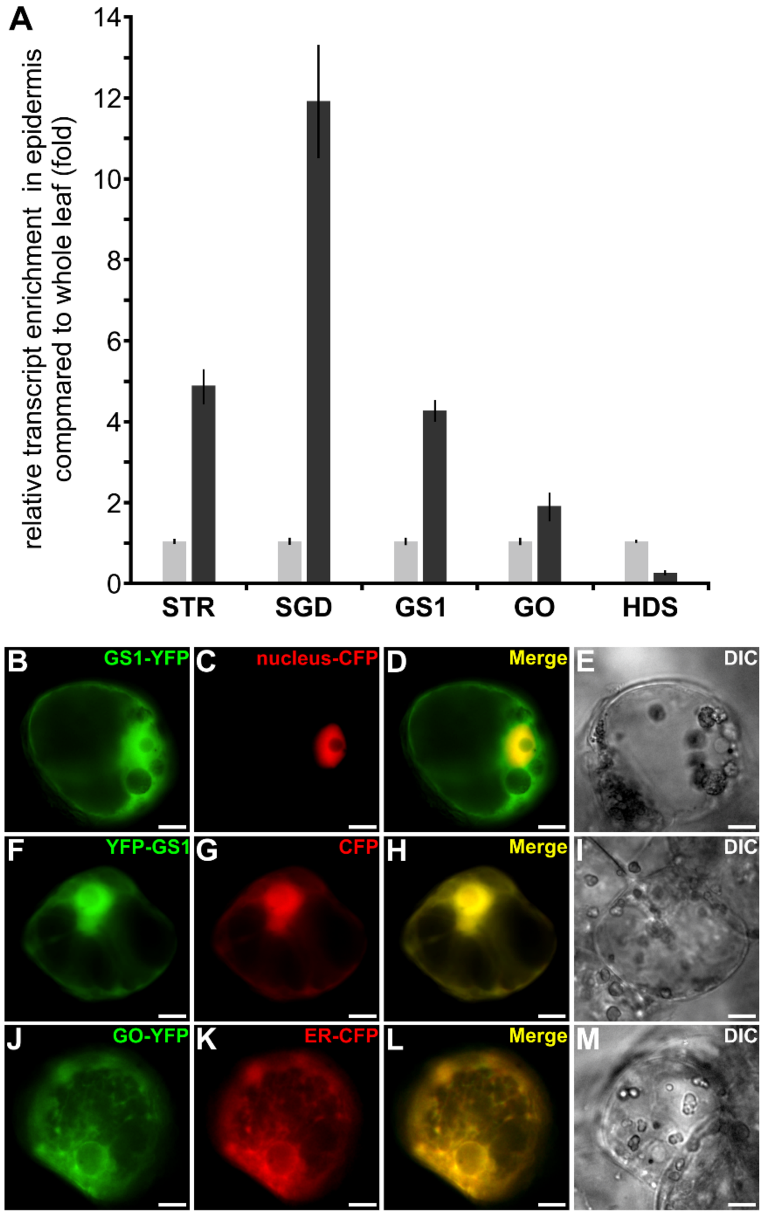

Supplementary Figure 26. GS1 and GO (CYP71D1V1) are located in close vicinity in planta. (A) GS1 and CYP71D1V1 transcripts co-localize with strictosidine synthase (STR) and SGD transcripts in *C. roseus* leaf epidermis. The relative expression of STR, SGD, GS1 and GO (CYP71D1V1) was evaluated in epidermis enriched fractions of leaves and compared to the whole leaf fraction. Expression of hydroxymethylbutenyl 4-diphosphate synthase (HDS) known to accumulate within internal phloem associated parenchyma was used as a control. Epidermis enriched transcript fractions were generated by a carborundum abrasion and both fraction types were retro-transcribed before determination of gene expressions by qPCR. Transcript copy numbers were normalized using CrRPS9 and expressed relative to the amount of transcript measured in the whole leaf fraction. Each assay was performed in triplicate, and expression measurements were performed at least twice with independent experimental replicates. (B-M) Subcellular localization of GS1 and GO (CYP71D1V1) YFP fusion proteins in *C. roseus* cells. Cells

were transiently co-transformed with plasmids expressing GS1-YFP (B), YFP-GS1 (F) or GO (CYP71D1V1)-YFP (J) and plasmids encoding a nuclear CFP marker (nucleus-CFP, C), a nucleocytosolic CFP marker (CFP, G) or an endoplasmic reticulum CFP marker (ER-CFP, K). Colocalization of the fluorescence signals appears in yellow when merging the two individual (green/red) false color images (D, H, L). Cell morphology is observed with differential interference contrast (DIC) (E, I, M). Bars, 10 μm.

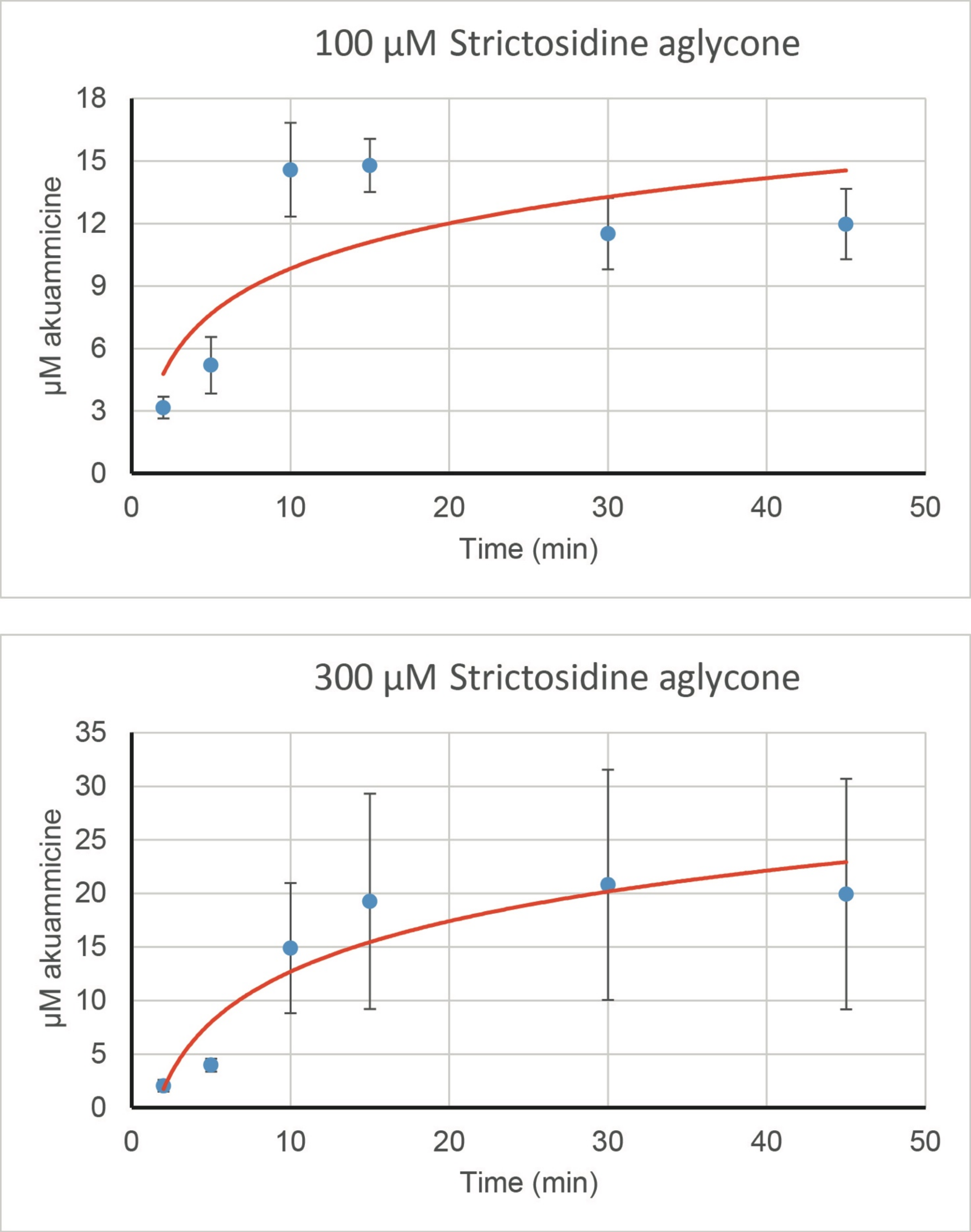

Supplementary Figure 27. Accumulation of akuammicine over time using different concentrations of substrate (strictosidine aglycone). Error bars in all cases represent the standard error of three replicates

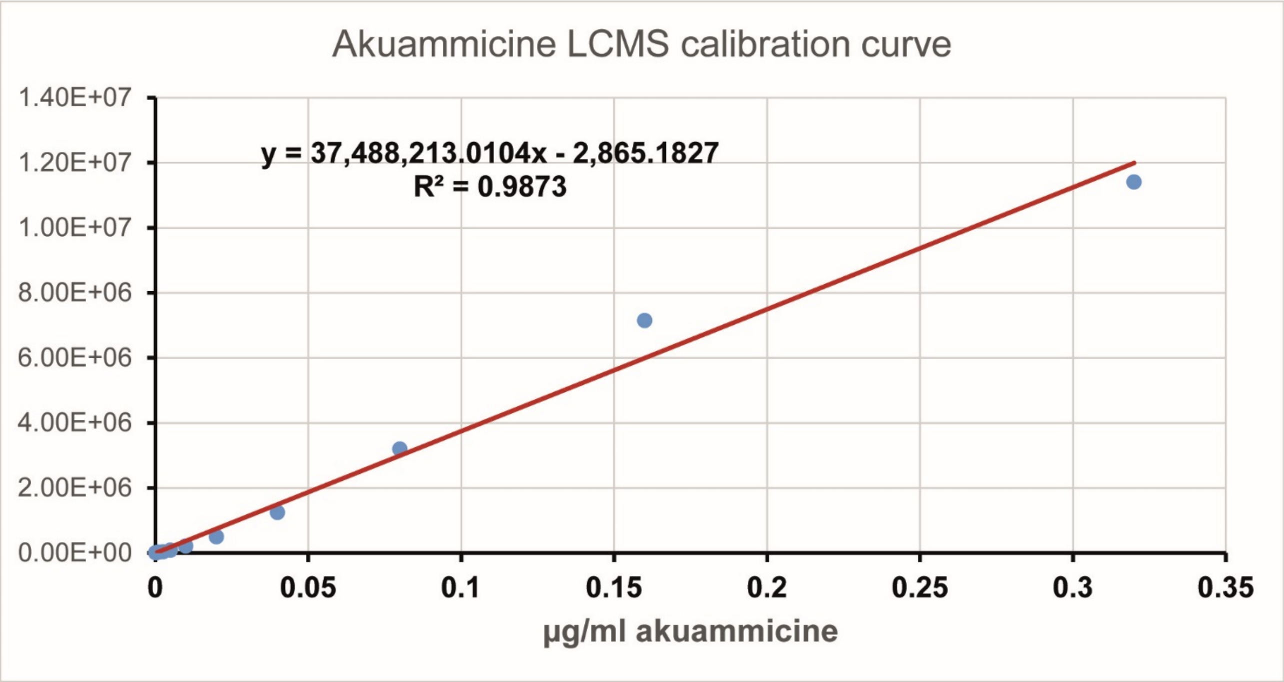

Supplementary Figure 28. Calibration curve for quantification of akuammicine by LCMS.

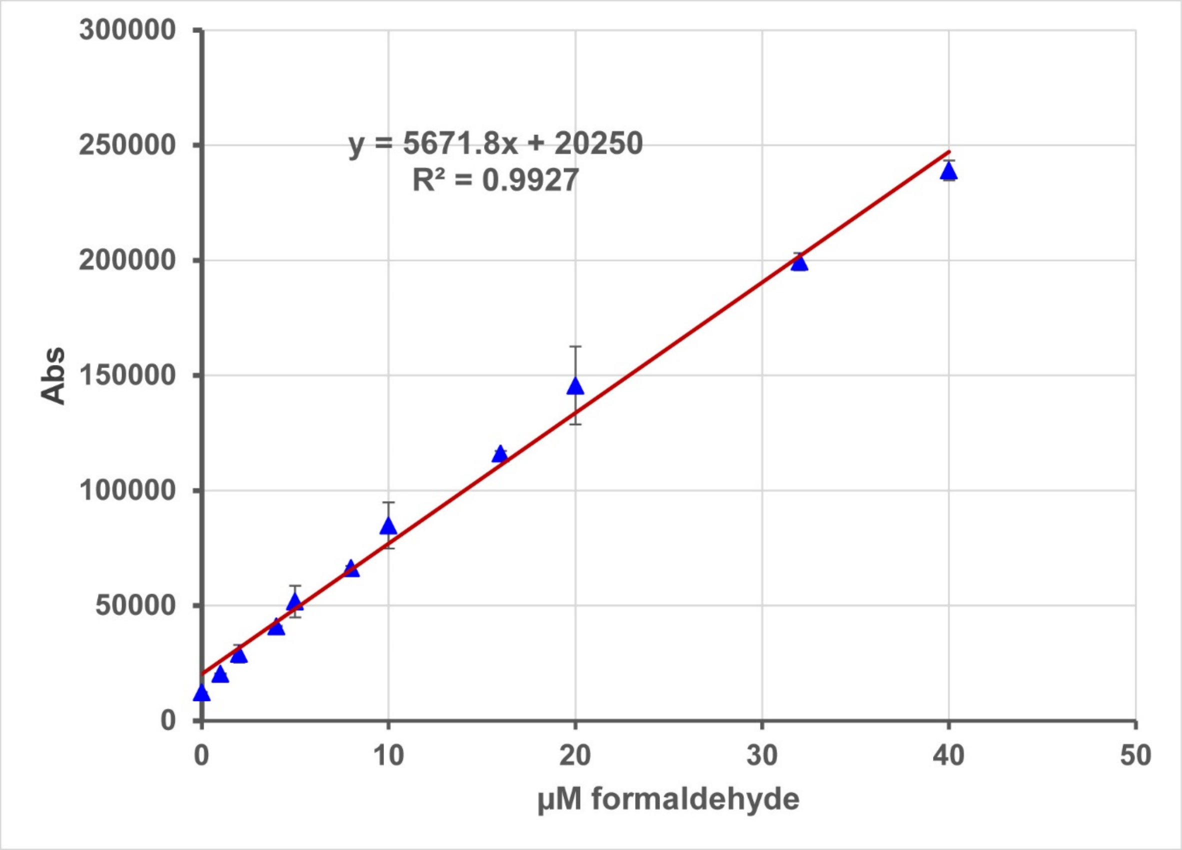

Supplementary Figure 29. Calibration curve for quantification of formaldehyde by fluorescence spectroscopy. Error bars in all cases represent the standard error of three replicates.
